# Objectively determined physical activity and adiposity measures in adult women: A systematic review and meta-analysis

**DOI:** 10.3389/fphys.2022.935892

**Published:** 2022-08-23

**Authors:** Yining Lu, Huw D. Wiltshire, Julien S. Baker, Qiaojun Wang, Shanshan Ying, Jianshe Li, Yichen Lu

**Affiliations:** ^1^ Faculty of Sport Science, Ningbo University,Ningbo, China; ^2^ Cardiff School of Sport and Health Sciences, Cardiff Metropolitan University, Cardiff, United Kingdom; ^3^ Centre for Health and Exercise Science Research, Department of Sport, Physical Education and Health, Hong Kong Baptist University, Kowloon, Hong Kong SAR, China; ^4^ Department of Sport and Physical Education, Zhejiang Pharmaceutical College, Ningbo, China

**Keywords:** accelerometer, pedometer, physical activity, adiposity, adult women

## Abstract

The prevalence of adiposity is increasing among adult women. Although emerging evidence suggest that all patterns of heightened physical activity (PA) are important to benefit adiposity, the relationship between objectively assessed intensities of PA and adiposity in women has not yet been assessed. Therefore, this systematic review and meta-analysis aims to qualitatively synthesize and quantitatively assess the evidence for any relationship between objectively measured PA and a wide range of adiposity indicators to guide PA prescription in adult women. Four databases (PubMed, Web of Science, Scopus, and the Cochrane library) were searched for eligible studies. 35 studies were included (25 observational and 10 interventional studies), with a total of 9,176 women from 20 countries included. The overall pooled correlation for random effects model (*n* = 1 intervention and *n* = 15 cross-sectional studies) revealed that the total volume of physical activity (TPA) was moderately associated with percentage body fat (%BF) (*r* = −0.59; 95% CI: −1.11, −0.24; *p* = 0.003). There was a weak but significant association between MVPA with body mass index (BMI), waist circumference (WC), and visceral adiposity. Daily steps were significantly associated with BMI, %BF, WC, and fat mass, with the strongest association with %BF (*r* = −0.41; 95% CI: −0.66, −0.19; *p* < 0.001). Walking programs resulting in increasing daily steps only had a significant effect on WC (SMD = −0.35; 95% CI: −0.65, −0.05; *p* = 0.02). Overall, objectively determined PA in terms of steps, TPA and MVPA were favorably associated with adiposity outcomes. The improvement in adiposity can be achieved by simply accumulating more PA than previously and adiposity is more likely to be benefited by PA performed at higher intensity. Nonetheless, these results should be interpreted with caution as there were a small number of studies included in the meta-analysis and the majority of studies included utilized cross-sectional designs.

## 1 Introduction

The prevalence of adiposity is one of the biggest concerns for global public health. Obesity has been significantly associated with the increased risk of type 2 diabetes, hypertension, cardiovascular diseases, certain cancers (Dwivedi et al., 2020; Julian et al., 2022; Wagner and Brath, 2012), and increased mortality (Whitlock et al., 2009). Adiposity indicates a state of positive energy balance. To deal with adiposity, a negative energy balance is required. Since physical activity (PA) is an important component of energy expenditure, many studies have examined the effect of PA on body weight management, and furthermore the related health outcomes. The Advisory Committee for the 2008 Physical Activity Guidelines reported that a minimum of 150 min moderate-to-vigorous intensity physical activities (MVPA) per week could contribute to 1–3% weight loss (Jakicic and Davis, 2011). Furthermore, an hour of moderate physical activity (MPA) is indicated to reduce the risk of adiposity by 19% among normal-weight and by 12% in overweight women (Rosenberg et al., 2013), and for substantial weight loss and adiposity improvement, a minimum of 300–420 min of MVPA weekly is needed (Johnson et al., 2021). On the contrary, several studies report limited weight loss after engaging in exercises due to the compensatory effects of other components of energy balance and gender differences in activity energy expenditures (Donnelly and Smith, 2005; Foright et al., 2018). Nonetheless, health benefits can be evoked with MVPA among both normal-weight and overweight or obese individuals irrespective of weight loss (Gaesser and Angadi, 2021; Johnson et al., 2021). Of note, the prediction of adiposity related health risk varies with the indicator used (Kahn et al., 2012; Tselha et al., 2019). Generally, overweight is defined as a body mass index (BMI) ≥ 25, and obesity as BMI ≥30. However, the measures of central adiposity such as waist circumference (WC) have been demonstrated as better indicators for detecting type 2 diabetes mellitus (Kapoor et al., 2020). Likewise, better predictive utility has been reported when using percentage body fat (%BF) and visceral adipose tissue (VAT) for metabolic health (Baudrand et al., 2013; Oliverors et al., 2014). Therefore, a wide range of adiposity indicators need to be taken into consideration when assessing its relationship with PA.

One the other hand, the majority of findings are based upon subjectively estimated PA (e.g., questionnaires and interviews) and focus on PA performed at least moderate intensity which preclude the ability to precisely determine the relationships between all patterns of PA and adiposity. Although there has been emerging evidence that light intensity physical activity (LPA) and non-bout PA are beneficial to health (Carson et al., 2013; Spittaels et al., 2012; Loprinzi, 2017; Marschollek, 2015), few studies have examined the association with adiposity using objective measured PA, which eliminates the measurement error with regard to lower intensity and sporadic PA. Despite the health benefits, 30.7% of adult women fail to meet the PA recommendations (Guthold et al., 2018). Furthermore, women suffer a consistently higher prevalence of adiposity than men, and have a greater increase of obesity prevalence, with 3.4% recorded in 1975 rising to 15% recorded in 2016 (Collaboration N.R.F., 2017; Kelly et al., 2008). Furthermore, women possess special biological, behavioral and socioeconomic characteristics which contribute to differences in attitude to physical activity patterns, and ultimately in exposure to increased risk of overweight or obesity (Herman et al., 2011; Althoff et al., 2017; Cooper et al., 2021; Kim and Singh, 2022). Moreover, age, race and menopausal status also influence the associations between PA and weight gain, %BF and fat distribution (Sims et al., 2012; Slater et al., 2021; Toth et al., 2000). Therefore, a better understanding of the association between PA and adiposity among women is important for preventing and improving increasing prevalence.

Given the paucity of investigations focusing on the relationship between all patterns of PA and a wide range of adiposity indicators, rigorous scientific assessments of these associations in adult women are needed. Therefore, the main purpose of this systematic review and meta-analysis was to qualitatively synthesize and quantitatively assess any associations between objectively determined PA and adiposity markers among adult women to guide PA prescription.

## 2 Methods

### 2.1 Protocol registration

The research protocol was registered in the International Prospective Register of Systematic Reviews (PROSPERO: registration number CRD42022307774). The systematic review and meta-analysis were conducted following guidelines outlined by the Preferred Reporting Items for Systematic Reviews and Meta-Analyses (PRISMA) (Moher et al., 2009).

### 2.2 Inclusion criteria and study selection

The Participants Interventions, Comparisons, and Outcomes (PICO) formatted research questions were used to clarify the inclusion criteria (Schardt et al., 2007).

#### 2.2.1 Participants

Apparently healthy women with a mean age range of 18–64 years were included in the study. Participants aged 18–39 and 40–64 were further stratified as young and middle-aged adults respectively (Murray et al., 2021).

Women with any conditions that tended to be a barrier to physical activity were excluded, including the presence of cardiovascular disease risk factors (e.g., hypertension, elevated fasting glucose, and dyslipidemia), diagnosed cardiovascular diseases (e.g., heart failure, serious arrhythmias, and peripheral vascular disease), physical or psychological disorders, previous stroke or myocardial infarction, diabetes (type 1 or type 2), previous surgery/banding, and chronic pain. Women who were pregnant, postpartum, or lactating, or were elite athletes were also excluded.

#### 2.2.2 Interventions (exposures)

PA assessed by accelerometers or pedometers were identified as exposures. For interventional studies, interventions were focused on physical activity exclusively with no other combined interventions, such as diet and supplements, which might affect health outcomes directly. Total energy expenditure obtained from double labelled water and calorimetry were not recognized as an included exposure because these were also affected by dietary intake and there were no daily or hourly data available for energy expenditure calculation (Sirard and Pate, 2001). Additionally, activity energy expenditure was not included due to the invalid estimation by accelerometers (Kossi et al., 2021).

#### 2.2.3 Comparisons

Studies reported the estimates in quantiles, and studies that treated PA variables as continuous measures were included. Various steps, volumes, frequencies, durations, and intensities of objectively measured PA were identified for comparisons.

#### 2.2.4 Outcomes

Five adiposity outcomes were included: 1) BMI, 2) %BF, 3) WC, 4) fat mass (FM), 5) visceral adipose tissue (VAT).

#### 2.2.5 Study design

Observational studies and intervention studies were included.

#### 2.2.6 Other criteria

Only literatures published in English and in peer-reviewed journals were included. Abstracts, conference proceedings, unpublished studies and grey literatures were excluded. Studies included participants of both sexes and were considered eligible when data for women were available. When literatures were from the same study, the one with the largest sample size, the longest total intervention period, or with the most detailed data set were used.

### 2.3 Information sources and search strategy

The electronic search strategy was guided by two researchers with expertise in systematic reviews. Four electronic databases including PubMed, Web of Science, Scopus and the Cochrane Library were searched from 1 January 1990 to 31 January 2022. Search terms were applied to titles and abstracts and combined with the keywords such as “objectively”, “physical activity”, “pedometer”, “accelerometry”, “body composition”, “overweight”, “obesity”, “adiposity”, “BMI”, “fat”, “waist”, and “fat mass”. The detailed search strategy is available in Supplementary Table A. Additionally, the literature list obtained was then manually searched to identify eligible references from included studies. Finally, search results were all imported into Endnote (Endnote 20, Wintertree Software Inc., China).

### 2.4 Data extraction

For each included study, descriptive data, exposure, finding, as well as information regarding confounders were extracted independently by two reviewers (Yining Lu and Qiaojun Wang) and inputted into Excel (Microsoft Corp.). Disagreements at any stage were resolved through discussion and all results were checked by a third reviewer (Shanshan Ying). The extracted data were: 1) reference details (e.g., first author, publication year, country); 2) study design, follow-up period (if applicable); 3) participants (e.g., sample size, sociodemographic characteristics); 4) protocol for PA assessment (e.g. device details, location, setting, required wearing time, valid wearing time); 5) PA measures (e.g., PA categories, definitions/cut-off points; 6) adiposity measures; 7) statistical analysis; 8) main findings (e.g., risk ratios, associations and differences in means). Statistically significant findings were identified when *p* < 0.05. Risk of bias and quality assessment.

The Newcastle-Ottawa Scale (NOS) was used to assess the risk of bias in nonrandomized studies (nonrandomized interventions and observational studies) (Wells et al., 2019). The NOS consisted of three components: selection, comparability, and outcome. A star was awarded for each question within the selection and outcome domains and a maximum of two stars was awarded for the comparability domain. For the comparability domain, we considered age to be the most important confounder due to its association with both PA and health. The maximum number of stars that a cross-sectional design and a longitudinal design could be awarded was seven and nine respectively. For cross-sectional designs, a total number of stars greater than or equal to 4 was defined as high quality, and below 4 was defined as low quality. The cut-off for longitudinal designs was 5 (Ramsey et al., 2022; Ramakrishnan et al., 2021). For RCTs, the Cochrane collaboration’s tool was used (Higgins et al., 2011), which comprised 6 domains with 7 questions, including: selection bias (random sequence generation and allocation concealment), performance bias (blinding of participants and personnel), detection bias (blinding of outcome assessment), attrition bias (incomplete outcome data), reporting bias (selective outcome reporting), and other sources of bias. The risk of bias for each question was judged as “low”, “unclear” or “high”, and finally, the overall quality was defined as high if all domains were low risk of bias.

The Grading of Recommendations Assessment, Development, and Evaluation (GRADE) was used to evaluate the quality of evidence for each PA measure (Guyatt et al., 2011). The quality of evidence was classified as high, moderate, low, and very low, with the evidence from randomized studies starting as high quality and the evidence from non-randomized or observational studies starting as low. Any discrepancy in rating was resolved by discussion and the results were verified by a third reviewer. Details of the risk of bias and quality assessment are presented in Supplementary Table B.

### 2.6 Statistical analysis

When there were more than 2 studies with comparable PA measures and adiposity indicators, a meta-analysis was planned. Regardless of the different cut-off points and definitions, LPA, MPA, vigorous intensity physical activity (VPA), MVPA and TPA were defined as reported in the studies. If studies measured physical activities in metabolic equivalents (METs), we used the cut-points proposed Ainsworth et al. (2011) (e.g., 1.6–2.9 METs was defined as LPA, 3–5.9 METs as MPA, and ≥6 METs as VPA).

When more than one statistical analysis was used, the following hierarchy was applied: 1) regression, 2) correlation, 3) ANOVA, 4) *t*-test/*U*-test/K-S test (Ramsey et al., 2022). When more than one adjusted model was used, the most adjusted model was applied (Aune et al., 2015).

Fisher’s z transformation was applied as recommended for correlational meta-analysis (Peterson and Brown, 2005). The standardized mean differences (SMD) were calculated using Hedges’ g. With regards to the effect size, according to Cohen’s recommendations, we classified the effect size as low (*r* = 0.1/SMD = 0.2), moderate (*r* = 0.3/SMD = 0.5), and high (*r* = 0.5/SMD = 0.8) (Cohen, 1988).

The random-effects model was used because of the high degree of heterogeneity among populations (age, BMI, ethics, and baseline PA). I^2^ statistics were used to measure heterogeneity among included studies, with I^2^ values of 25, 50 and 75% being categorized as low, moderate, and high, respectively (Higgins et al., 2003). Subgroup analyses were performed to identify the potential sources of heterogeneity from five aspects, including age (young/middle-age), overweight/obese (BMI<25/BMI≥25), menopausal status (postmenopausal/premenopausal), country, and ethnicity. Publication bias was assessed using Egger’s test and funnel plots for PA category for at least ten studies (Egger et al., 1997). Finally, we conducted sensitivity analysis by removing studies with low quality.

Statistical analyses were performed with Review Manager (RevMan), version 5.4.1 (The Cochrane Collaboration, 2017).

## 3 Results

### 3.1 Study identification and selection

**FIGURE 1:**
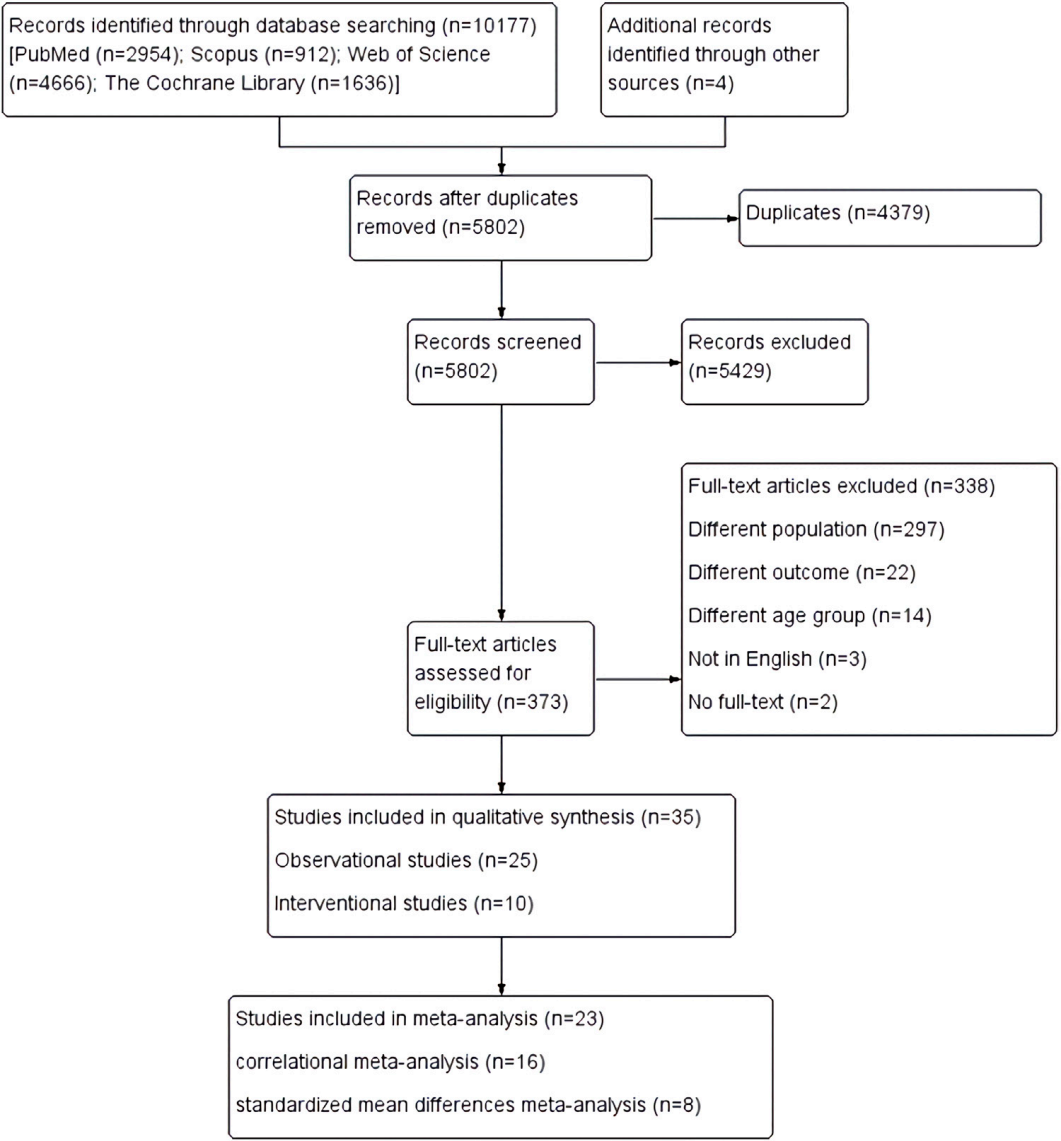
PRISMA process of study selection.

A total of 10,177 records were identified from the database search between 1 January 1990 and 31 January 2022 from PubMed, Scopus, Web of Science, and the Cochrane Library. Additionally, 4 records were identified through reference list screening. After removing the duplicate records (*n* = 4,379), 5,802 studies were further screened based on title and abstract. Exclusion of irrelevant studies resulted in 373 records. After examining the full text, finally, 35 eligible studies were included in the present review. The processes of study selection followed the PRISMA guidelines and are presented in Figure 1.

### 3.2 Study characteristics

Out of 35 studies, 25 were observational studies, with 24 using a cross-sectional design and 1 longitudinal design (20-months follow-up), reporting cross-sectional findings (Bailey et al., 2007). Detailed characteristics of observational studies are illustrated in Table 1. 10/35 were intervention studies (RCTs: 3 and non-RCTs: 7), with the intervention length ranging from 9 weeks (Hasan et al., 2018) to 24 weeks (Bailey et al., 2019; Holliday et al., 2018; Moreau et al., 2001). A detailed description of the intervention studies is presented in Table 2.

**TABLE 1:** Characteristics of observational studies.

References	Study design	Country	Sample size	PA measure	Health outcome	Association
**Ayabe et al. (2013)**	cross-sectional	Japan	42	A: Lifecorder-Ex; uniaxial. . Duration (min/d), frequency (bouts/d). MVPA, MVPA bout (32s, 1, 3, 5min); VPA, VPA bout (32s, 1, 3, 5min)	**VAT,** by computed tomography (CT)	Regression: 1) Frequency of MVPA bout (1min,3min), TPA bout (3min, 5min) were favorable associated with VAT; 2) Duration of MVPA bout (1min) was favorable associated with VAT; 3) VPA was favorable associated with VAT; 4) No significant relationship between MVPA and VAT
**Bailey et al. (2015)**	cross-sectional	United States	343	A: ActiGraph GT3x; triaxial, Duration (min/d): LPA, MPA, VPA; Intensity of PA (METs)	**%BF; Obese (%BF ≥ 32%)** by BODPOD	Regression: adjusted for age and seasonality. 1) Duration of MPA, VPA was favorably associated with %BF, LPA had no association with %BF; 2) For every 10 min spent in MVPA per day, the odds of being obese reduced by 29%. 3) For PA intensity (≤4 METS), %BF decreased as duration increased; For PA intensity (≥4.9 METS), no benefit of accumulating more than 30 min per week. ANOVA:1) Group (VPA ≥30min/w) had significantly lower %BF than Group (VPA <30min/w); 2) Group (MVPA ≥30 min/d) had significantly lower %BF than Group (MVPA <30 min/d); 3) Group (MVPA ≥90 min/d) was associated with the lowest %BF
**Bailey et al. (2014)**	cross-sectional	United States	186	P: Omeron HJ-720-ITC. Steps (n/d) Aerobic steps (n/d) (60/min for a minimum of 10 min)	**%BF, WC, BMI; Overweight (25≤BMI<30), Obese (%BF ≥ 32% or BMI≥30)** by BODPOD	Regression: adjusted for age, average daily temperature, and menstrual cycle. 1) Every increase of 1,000 steps/d was associated with a 2.4% lower %BF; 2) For every increase of 1,000 steps/d, the odds of being obese reduced by 22%; 3) For Group (≥10,000 steps/d), the odds of being overweight reduced by 65% and the odds of being obese were reduced by 80% compared with Group (<10,000 steps/d)Correlation:1) Steps/d was favorably associated with %BF; has no association with WC, BMI; 2) Aerobic steps/d has no association with body composition index
**de Hoed and Westerter, (2008)**	cross-sectional	Netherlands	80	A: Tracmor IV, triaxialTPA (Mcnts/d)	**%BF** by underwater weighing	Regression: adjusted for BM, height, and seasonality. 1) TPA was favorably associated with %BF
**Diniz et al. (2015)**	cross-sectional	Brazil	49	A: ActiGraph GT3x; triaxial. Duration (min/w): MVPATPA (counts/min) meet/not meet MVPA (Duration)	**%BF, BMI** by dual-energy X-ray absorptiometry (DXA)	U-test: 1) Group (MVPA <150 min/w) had a higher %BF compared to (MVPA ≥150 min/w); 2) No differences in BMI between Group (MVPA <150 min/w) and Group (MVPA ≥150 min/w); 3) Group (NAF) had a higher TPA compared to Group (HAF); 4) No differences in %MVPA between Group (NAF) and Group (HAF)
**Graff et al. (2012)**	cross-sectional	Brazil	68	P: BP 148. Steps (n/d)	**%BF, BMI, WC** by calculation	t-test and U-test: 1) Group (Steps/d < 6,000) had higher BMI, WC, %BF than Group (Steps/d ≥ 6,000)
**Green et al. (2014)**	cross-sectional	United States	50	A: ActiGraph GT3X+; triaxial. Duration (min/d): LPA, MVPA; Duration (min/w): MVPA bout (10min)	**WC** by anthropometric tape	Correlation: adjusted for SB, VO2peak, and BM. 1) LPA, MVPA and MVPA bouts had no association with WC
**Hornbuckle et al. (2005)**	cross-sectional	United States	69	P: New Lifestyles Digi-Walker SW-200. Steps (n/d)	**BMI, %BF, WC** by BODPOD	Correlation: adjusted for age and caloric intake. 1) Steps/d was favorably associated with BMI, %BF, WC
**Koniak-Griffin et al. (2014)**	cross-sectional	United States	210	A: Kenz Lifecorder Plus; uniaxial. Duration (min/d): MVPA, MVPA bout (10min) Steps (n/d)	**BMI, WC** by calculation and anthropometric tape	Correlation: 1) Steps/d was favorably associated with BMI, WC; 2) MVPA was favorably associated with WC; had no association with BMI; 3) MVPA bouts had no association with BMI, WC
**Panton et al. (2007)**	cross-sectional	United States	35	P: Yamax Digi-Walker SW-200, sealed. Steps (n/d)	**BMI, WC** by calculation and anthropometric tape	Correlation: 1) Steps/d was favorably associated with BMI; had no association with WC.ANOVA:1) Group (Steps/d < 5,000) had higher BMI compared to Group (Steps/d ≥ 5,000); 2) No differences in WC between Groups
**Park et al. (2011)**	cross-sectional	Japan	100	A: Lifecorder EX, uniaxial. Duration (min/d): LPA, MPA, VPASteps (n/d)	**BMI, FM, %BF** by underwater weighing	Regression: 1) Steps/d was favorably associated with BMI, FM and %BF; 2) LPA had no association with BMI, %BF or FM; 3) MPA was favorably associated with BMI and FM; had no association with %BF; 4) VPA was favorably associated with BMI, FM and %BF
**Pelclová et al. (2012)**	cross-sectional	Czech Republic	167	A: ActiGraph GT1M, uniaxial. Duration (min/w):MPA meet MPA/do not meet MPA (Duration, Frequency)Steps (n/d)	**BMI, FM, VAT** by bioelectrical impedance	Correlation: 1) MPA and steps/d were favorably associated with BMI, FM, and VAT; ANOVA: 1) Group (150 ≤ MPA≤300min/w) had higher FM than Group (MPA>300min/w); no differences in BMI and VAT between groups; 2) Group (not meet MPA 5 × 30 min/week) had higher BMI, FM, VAT than Group (meet MPA 5 × 30 min/week); 3) The frequency of performing 10,000 steps/day in a week was favorably associated with BMI, FM, VAT
**Phelan et al. (2007)**	cross-sectional	United States	237	A: RT3; triaxial. Duration (min/d): LPA, MPA, VPA, MVPA	**BMI** by calculation	K-S test: 1) MVPA was higher in Group (weight-loss-maintainer) than Group (always-normal-weight); 2) VPA was higher in Group (weight-loss-maintainer) than Group (always-normal-weight); 3) no differences in MPA and LPA between groups. 4) Most Group (always-normal-weight) engaged in 30–60 MVPA min/d; 5) Most Group (weight-loss-maintainer) engaged in >60 MVPA min/d
**Slater et al. (2021)**	cross-sectional	New zealand	275	A: ActiGraph w-GT3X, Acti-Watch; triaxial. Duration (min/d): MVPATPA (cpm/d)	**%BF, WC, BMI, VAT** by DXA	Regression: adjusted for age and socioeconomic level. 1) TPA was favorably associated with %BF and VAT; had no association with WC or BMI; 2) MVPA was favorably associated with %BF, VAT, WC and BMI
**Smith et al. (2013)**	cross-sectional	United States of America	201	A: Actigraph AM7164, uniaxialDuration (min/d): LPA, MVPATPA (min/d ≥ 100 counts)	**VAT** by CT	Regression: adjusted for age, BMI, race-ethnicity, education, SB1) LPA, MVPA had no association with VAT; 2) TPA was favorably associated with VAT
**Sternfeld et al. (2005)**	cross-sectional	United States	248	A: CSA; uniaxial. Duration (min/d): MPA, VPA; TPA (cpm/d)	**%BF, WC** by DXA	Regression: age, menopausal status, education, and health status. 1) TPA and MPA had no association with %BF or WC in both ethic groups; 2) VPA was favorably associated with %BF and WC in Group (White); had no association in Group (Chinese)
**Strath et al. (2008)**	cross-sectional	United States	1,594	A: AM-7164; uniaxial. Duration (min/d): MVPA bout (10min), MVPA non-bout	**BMI, WC** by calculation and anthropometric tape	Regression: adjusted age, age-squared, race/ethnicity, smoking, and health status.1) MVPA bouts was favorably associated with BMI and WC; 2) MVPA non-bouts had no association with BMI and WC; 3) The strength of association with decreased BMI was nearly 7 times greater for MVPA bout than for MVPA non-bouts; 4) The strength of association with decreased WC was nearly 5 times greater for MVPA bout than for MVPA non-bouts
**Thompson et al. (2004)**	cross-sectional	United States	80	P: Yamax Digi-Walker SW-200. Steps (n/d)	**BMI, %BF, WC** by BODPOD	Correlation: adjusted for age. 1) Steps/d was favorably associated with BMI, %BF, WC; ANOVA:1) Group (<6000steps/d) had higher BMI than Group (6,000–9,999) and Group (≥10,000); 2) Group (≥10,000) had the lowest %BF, and WC among three groups
**Tolonen et al. (2018)**	cross-sectional	Finland	837	P: Walking style One, HJ-152R-E Steps (n/d)	**BMI** by calculation	ANOVA: 1) Group (steps/d > 8,765) had lower BMI than Group (Steps/d < 6,317)
**Tucker and Peterson, (2003)**	cross-sectional	United States	278	A: CSA; uniaxial. TPA (counts) (min/w). Intensity and duration of PA (counts/10min)	**%BF** by BODPOD	Regression: adjusted for BM, and energy intake. 1) TPA was favorably associated with %BF; 2) The intensity and duration were favorably associated with %BF.
**Tudor-Locke et al. (2009)**	cross-sectional	Australia	158	P: Yamax Digiwalker SW700. Steps (n/d)	**BMI** by calculation	ANOVA: 1) Group (Fewer Steps/day) had higher BMI.
**Vella et al. (2011)**	cross-sectional	United States	60	A: Actigraph GT1M; uniaxial meet MVPA/do not meet MVPA (duration)	**BMI, FM, %BF, WC** by BODPOD	t-test: 1) No differences in BMI, FM, %BF or WC between groups
**Vella et al. (2009)**	cross-sectional	United States	60	A: Actigraph GT1M; uniaxial Steps (n/d)	**WC** by anthropometric tape	correlation: 1) Steps/d had no association with WC
**Van Dyck et al. (2015)**	cross-sectional	multi-country	3,027	A: ActiGraph 7,164/71,256, GT1M, ActiTrainer, GT3X; uniaxialandtriaxial. Duration (min/d): MVPATPA (cpm/d)	**BMI** by calculation	Regression: socio-demographic status and accelerometer wear time. 1) MVPA and TPA was favorably associated with BMI with country-specific associations
**Bailey et al. (2007)**	cohort study/cross-sectional	United States	228	A: ActiGraph; uniaxial The LPA, MPA, VPA groups based on the average of the highest 7 epochs (10min); The increased-, maintained- and decreased- intensity groups based on changes of PA intensity group from baseline to follow-up	**%BF** by BODPOD	ANCOVA: adjusted for age and TPA. 1) Group (VPA) had lower BF% than Group (MPA) or Group (LPA); 2) no difference in BF% between Group (MPA) and Group (LPA); 3) Group (decreased intensity) had a higher proportion of having a higher %BF at follow-up than Group (maintained intensity) and Group (increased intensity)

**Note:** A: accelerometer; BMI, body mass index; FM, fat mass; HAF, high abdominal fat; METs, metabolic equivalents; MPA, moderate intensity physical activity; MVPA, moderate to vigorous intensity physical activity; NAF, normal abdominal fat; P, pedometer; PA, physical activity; SB, sedentary behaviors; TPA, total physical activity; VAT, visceral adipose tissue; VPA, vigorous physical activity; WC, waist circumference; %BF, percentage body fat.

**TABLE 2:** Characteristics of intervention studies.

References	Study design	Country	Sample size	Intervention	PA measure	Health outcome	Association
**Bailey et al. (2019)**	random experiment	United States	92	24-weeks incremental walking program; Group 1 (*n* = 34): asked to walk 10,000 steps/d, 6 days/week; Group 2 (*n* = 34): asked to walk 12,500 steps/d, 6 days/week; Group 3 (*n* = 24): asked to walk 15,000 steps/d, 6 days/week	A: ActiGraph GT3x; triaxial. P: Omeron HJ-720-IT; Duration (min/d): LPA, MVPA. Steps, Aerobic steps (n/d)	**BMI, %BF, FM, VAT (mass, volume)** by DXA	ANOVA: 1) Steps/d increased from baseline in all three Groups, with a significant intervention effect between groups; 2) LPA increased from baseline only in Group 3, with no effect for groups; 3) MVPA and aerobic steps/d increased from baseline in all three Groups, with a significant main effect for groups between Group 2&3 and Group 1; 4) BMI increased from baseline only in Group 1; 5) %BF and VAT mass and volume had no change in all three groups; 6) FM increased from baseline in Group 2&3; 7) no follow-up differences in all parameters between groups
**Cayir et al. 2015**	randomized controlled trial	Turkey	84	3-months pedometer-based walking program. Group 1 (n = 45): with pedometer. CONT (n = 39): without pedometer	P: Voit 3 days. Steps (n/d)	**BMI, %BF, WC** by anthropometry	ANCOVA: baseline BM, BMI, %BF. 1) BMI, %BF, WC decreased in Group 1; had no change in CONT; 2) ΔBMI, Δ%BF, ΔWC were larger in Group 1 compared to CONT
**Hasan et al. (2018)**	Quasi-experimental design	UAE	52	9-weeks pedometer-based walking program, asked to walk 10,000 steps per day	P: KenzLifeCoder e-step. Steps (n/d)	**BMI, %BF, VAT (area), FM, WC** by InBody	Correlation: 1) After intervention, steps/d was favorably associated with BMI, FM, and %BF; had no association with VAT or WC t-test:1) After intervention, FM, BMI, %BF, VAT, WC decreased; 2) In Group (18 ≤ BMI<25), after intervention, BMI and FM decreased; no changes in %BF, VAT or WC; 3) In Group (BMI≥25), after intervention, BMI, FM, %BF, WC, and VAT decreased
**Holliday et al. (2018)**	randomized controlled trial	United Kingdom	58	24-weeks PA interventionGroup 1 (*n* = 20): asked to undertake 5 × 30 min of moderate-intensity exercise/week; Group 2 (*n* = 22): a points score allocated per 10-min of activity, Participants were instructed to accumulate 30 points per week, equating to 5 × 30 min of brisk walking; CONT (*n* = 16): asked to maintain their current lifestyle	A: ActiGraph GT3X+; triaxial %duration of LPA, MVPA	**FM, VAT (area), WC** by DXA	ANOVA: 1) Changes in WC was significantly greater in Group 2 at 24 weeks, compared with CONT; 2) There was a trend for greater reductions in FM in Group 2 vs. CONT (*p* = 0.075); 3) There was a trend for greater reductions in VAT in Group 2 vs. CONT (*p* = 0.053); 4) Parameters were unchanged in Group 1
**Hornbuckle et al. (2012)**	random experiment	United States	44	12-weeks exercise interventionGroup 1: asked to walk 10,000 steps/dGroup 2: asked to walk 10,000 steps/d + RT 2days/w (3 sets of 8–12 repetitions of 10 resistance exercises for the lower and upper body)	P: New Lifestyles Digi-Walker SW-200 Steps (n/d)	**BMI, WC, %BF, FM** by DXA	ANOVA: 1) Steps/d increased from baseline in both groups; 2) No changes in all parameters after intervention in Group 1; 3) WC, %BF and FM decreased after intervention in Group 2
**Moreau et al. (2001)**	randomized controlled trial	United States	24	24-weeks pedometer-based walking programGroup 1: provided with a target number of steps that would lead to a 3-km increase in daily walking; CONT: maintain current physical activity and subsequently wore a pedometer 1 week each month to document their walking	P: Yamax SW200 pedometer Steps (n/d)	**%BF** by BODPOD	ANOVA: 1) Steps/d increased from baseline in Group 1 compared with CONT; 2) %BF had no change in either group after intervention
**Musto et al. (2010)**	Quasi-experimental design	United States	77	12-weeks incremental walking program; asked to increase steps/d by 10% per week; the progression was reduced to a 3% when steps/d reached 10,000Group 1: improved steps/d by 3,000 or greater; CONT: stopped participating or did not achieve step improvement level	P: Sportline 330 Steps (n/d)	**BMI, WC** by calculation	ANOVA: 1) BMI decreased after intervention in Group 1 compared with CONT; 2) WC decreased after intervention in Group 1
**Pal et al. (2011)**	random experiment	Australia	28	12-weeks walking program; Group 1: asked to undertake 30 min of walking/day; with sealed pedometerGroup 2: asked to accumulate 10,000 steps/d, with unsealed pedometer	P: Yamax Digi-Walker SW-200 Steps (n/d)	**BMI, %BF, WC** by BIA	ANOVA: 1) Steps increased in both groups after intervention with between-group effects.2) no changes in BMI, %BF, WC in either group after intervention
**Sugawara et al. (2006)**	random experiment	Japan	17	12-weeks cycling training. Group 1 (*n* = 8): 180–300 kcal/session, 3–5 sessions/week at 40% HRR Group 2 (n = 9): at 70% HRR	A: Lifecorder; uniaxial Intensity LPA (<4METs), MPA (4-6METs), VPA (>6METs) (min/d)	**BMI** by calculation	ANOVA: 1) BMI decreased after intervention in Group 2
**Swartz et al. (2003)**	Quasi-experimental design	United States	18	4-weeks control period followed by 8-weeks walking program	P: Yamax Digi-Walker SW-200 Steps (n/d)	**BMI, %BF, WC** by BODPOD	ANOVA: 1) Steps/d increased during intervention period; 2) BMI, %BF, and WC had no change

**Note:** A: accelerometer; BMI, body mass index; CONT, control group; FM, fat mass; HRR, heart rate reverse; METs, metabolic equivalents; MPA, moderate intensity physical activity; MVPA, moderate to vigorous intensity physical activity; P, pedometer; PA, physical activity; RT, resistance training; SB, sedentary behaviors; TPA, total physical activity; VAT, visceral adipose tissue; VPA, vigorous physical activity; WC, waist circumference; %BF, percentage body fat.

Among observational studies, linear regression was the most used method to assess any associations (10/25), correlation analysis was used in 8 studies, ANOVA was used in 8 studies, and the *t*-test/*U*-test/K-S test was used in 4 studies. While in intervention studies, ANOVA was commonly used (9/10), with 1 study also including correlation analysis (Cayir et al., 2015). The rest one used a combination of *t*-test and correlation (Hasan et al., 2018).

Dietary intakes were analyzed in 4/35 observational studies (Graff et al., 2012; Hornbuckle et al., 2005; Park et al., 2011; Tucker and Peterson, 2003) and 8/10 intervention studies (Bailey et al., 2019; Hasan et al., 2018; Holliday et al., 2018; Hornbuckle et al., 2012; Moreau et al., 2001; Musto et al., 2010; Pal et al., 2011; Swartz et al., 2003).

### 3.3 Sample characteristics

The total sample size was 9,176, ranging from 17 (Sugawara et al., 2006) to 3,027 (Van Dyck et al., 2015) participants. The mean age of the women ranged from 18.2 (Bailey et al., 2019) to 64.2 (Pelclová et al., 2012) years. 11/35 studies focused on young female adults, while 21/35 studies focused on middle-aged women. 8/35 studies reported menstrual status, with 2 studies focusing on post-menstruation (Moreau et al., 2001; Diniz et al., 2015), 6 including pre-menstruation (Graff et al., 2012; Green et al., 2014; Slater et al., 2021; Sternfeld et al., 2005; Tucker and Peterson, 2003; Bailey et al., 2007). Moreover, 10/35 studies focused on overweight/obese females and 1 study consisted of a sample of weight-loss-maintainers (Phelan et al., 2007). 10/35 studies described the lifestyle of participants as physically inactive. 14/35 studies reported smoking status, with 8 studies utilizing participants who never smoked, 4 studies reported participants refrained from tobacco in the last 6 months, and 2 studies included mostly non-smokers (80–83%). Regarding education level, 8/35 studies indicated the percentage of participants who attended college or university, ranging from 37–100%. Social-economic levels were presented as low-income in 3 studies (Koniak-Griffin et al., 2014; Panton et al., 2007; Slater et al., 2021), college students in 4 studies (Hasan et al., 2018; Bailey et al., 2019; Bailey et al.,. 2014; de Hoed and Westerterp, 2008), the Third Age University students in 1 study (Pelclová et al., 2012), part-time employees in 1 study (Ayabe et al., 2013), and full-time employees in 2 studies (43–63%) (Tudor-Locke et al., 2009; Cayir et al., 2015).

Included studies were conducted in 11 countries. 4/35 studies included a sample from Asia (i.e., Japan, Turkey, and UAE), 4/35 from Europe (i.e., UK, Netherlands, Czech Republic, and Finland), 20/35 from North America (i.e., United States), 2/35 from South America (i.e., Brazil), and 3/35 from Oceania (i.e., New Zealand and Australia). Additionally, 1 study included a sample from 12 different countries (Van Dyck et al., 2015). Moreover, 20/35 studies reported the race of participants, with 9 studies including Caucasian mainly (73–96%), 4 studies included Asian (Ayabe et al., 2013; Park et al., 2011; Sternfeld et al., 2005; Sugiura et al., 2002), 3 studies included African American (Hornbuckle et al., 2005; Panton et al., 2007; Hornbuckle et al., 2012), and others reported ethnicities including Hispanic (Vella et al., 2011; Vella et al., 2009), Latina (Koniak-Griffin et al., 2014) and Pacific women (Slater et al., 2021). Detailed characteristics of participants are illustrated in Supplementary Table C.

### 3.4 Physical activity assessment

21/35 studies used accelerometers, with 1 study also using a pedometer (Bailey et al., 2019). 14/35 studies used pedometers. The intensity of PA was categorized using various cut-points. Detailed ascertainment and measurement characteristics of objectively measured PA are illustrated in Supplementary Table D.

Most studies (19/35) assessed daily steps, with 2 studies including aerobic steps (Bailey et al., 2019; Bailey et al., 2014). Seven studies assessed TPA, 7 studies measured the duration of LPA, 7 studies included MPA, 5 studies included VPA, and 10 studies included MVPA. Four studies examined PA in bouts (Ayabe et al., 2013; Green et al., 2014; Koniak-Griffin et al., 2014; Strath et al., 2008). Furthermore, 4 studies examined PA intensity (Bailey et al., 2015; Bailey et al., 2007; Tucker and Peterson, 2003; Sugawara et al., 2006), 2 studies evaluated the frequency of PA (Ayabe et al., 2013; Pelclová et al., 2012), and 3 studies examined the adherence to PA guidelines (Pelclová et al., 2012; Vella et al., 2011; Diniz et al., 2015).

### 3.5 Physical health outcome assessment

Of the 35 included studies, 23/35 studies reported BMI, 20/35 examined %BF, 18/35 measured WC, 7/35 investigated FM, and 6/35 included VAT.

### 3.6 Risk of bias assessment and the quality of evidence

Details of the risk of bias assessment for included studies is reported in Supplementary Table B 1&2. Out of 28 observational and non-RCTs design, 19 were categorized as high quality and 9 as low quality. 14/29 studies did not control for age, which was the most important covariate that we deemed for quality assessment. Among 7 RCTs, 3 were of high quality and 4 were unclear. The lack of presenting random sequence generation was the most common reason for risk of bias.

Moreover, according to the GRADE framework, very low to moderate quality of evidence were reported, with no upgrades. Supplementary Table B 3 outlines the details of the quality of evidence by study design and the PA measures.

### 3.7 Qualitative synthesis of associations between physical activity and adiposity outcomes

Adiposity variables were reported as BMI, %BF, WC, FM, and VAT, which were objectively measured by bioelectrical impedance, underwater weighting, computed tomography (CT), BODPOD, dual-energy X-ray absorptiometry (DXA) or calculation based on objectively measured variables. A favorable association or effect was considered when increased PA resulted in improved adiposity indicators or vice versa. An unfavorable association or effect was considered when increased PA resulted in poorer adiposity indicators or vice versa.

For a total of 10 interventional studies, 8 studies investigated the effects of participating in a long-term walking program on BMI (*n* = 7), %BF (*n* = 7), WC (*n* = 6), FM (*n* = 3) and VAT (*n* = 2). Five studies (2 quasi-experiment and 3 random experiment) reported a significant improvement in at least one adiposity measure (Cayir et al., 2015; Moreau et al., 2001; Musto et al., 2010; Bailey et al., 2019; Hasan et al., 2018), while 3 studies (1 quasi-experiment and 2 random experiment) reported no significant changes (Hornbuckle et al., 2012; Pal et al., 2011; Swartz et al., 2003). One random experiment examined the effects of MPA and VPA on BMI and reported a decrease in BMI only after participating VPA (Sugawara et al., 2006). One randomized controlled trial reported that there were no significant improvements on any adiposity variables after engaging in a 24-weeks moderate intensity exercise protocol at a volume of 30 min per day for 5 days per week. (Holliday et al., 2018).

Among 25 observational studies, 11 cross-sectional studies assessed the association between daily steps and adiposity measures. There were consistent results across studies of a favorable association with %BF, FM, and VAT. Evidence pertaining the association with BMI was equivocal, with 9/10 studies reporting a favorable association and 1/10 indicating null association in college female students (Bailey et al., 2014). The most inconsistent evidence was shown for WC, with 3/7 studies finding a beneficial association and 4/7 reported null. Seven studies evaluated the association between TPA and adiposity, with 6 (85.7%) reporting a favorable association. TPA was consistently reported to be favorably associated with VAT and to have no association with WC. While equivocal evidence was found for BMI and %BF.

### 3.8 Influence of physical activity intensity, duration, and frequency on adiposity outcomes

In observational studies (*n* = 5), LPA had no influence on adiposity. A total of 5 observational studies focused on MPA and there were consistent findings of favorable associations with FM and VAT, and no association with WC. The equivocal result was found for %BF, with a beneficial association reported in 1/3 studies and no association found in 2/3 studies. Additionally, 2/3 studies found that MPA was favorably associated with BMI and the remaining one study reported no differences between weight-loss-maintainer and always-normal-weight women. VPA was found to be beneficially associated with all adiposity outcomes, with 1 study reporting ethnic-specified association that favorable relationships between VPA with %BF and WC were not observed among Chinese women (Sternfeld et al., 2005). With regards to MVPA, findings were particularly inconsistent with adiposity outcomes such as VAT, %BF, BMI, and WC. Findings that the intensity of PA was significantly associated with adiposity outcomes were evidenced in experimental (Sugawara et al., 2006), longitudinal (Bailey et al., 2007) and cross-sectional studies (Tucker and Peterson, 2003; Bailey et al., 2015).

For PA in bouts, 3 studies investigated the association between 10-min MVPA bouts with BMI or WC, with 2 studies (Green et al., 2014; Koniak-Griffin et al., 2014) reporting null association and 1 study reporting a favorable association (Strath et al., 2008). One study reported that MVPA bouts lasting more than 1 min was beneficially associated with VAT (Ayabe et al., 2013). Furthermore, a favorable association between PA frequency and adiposity was found in all included studies based on cross-sectional evidence (Pelclová et al., 2012; Ayabe et al., 2013).

Finally, 3 studies examined the effect of PA recommendations for engaging in at least 150 min MVPA a week, with 1 study reporting a favorable association with %BF (Diniz et al., 2015) and the other 2 reporting no association (Vella et al., 2011; Pelclová et al., 2012).

### 3.9 Meta-analysis

**FIGURE 2:**
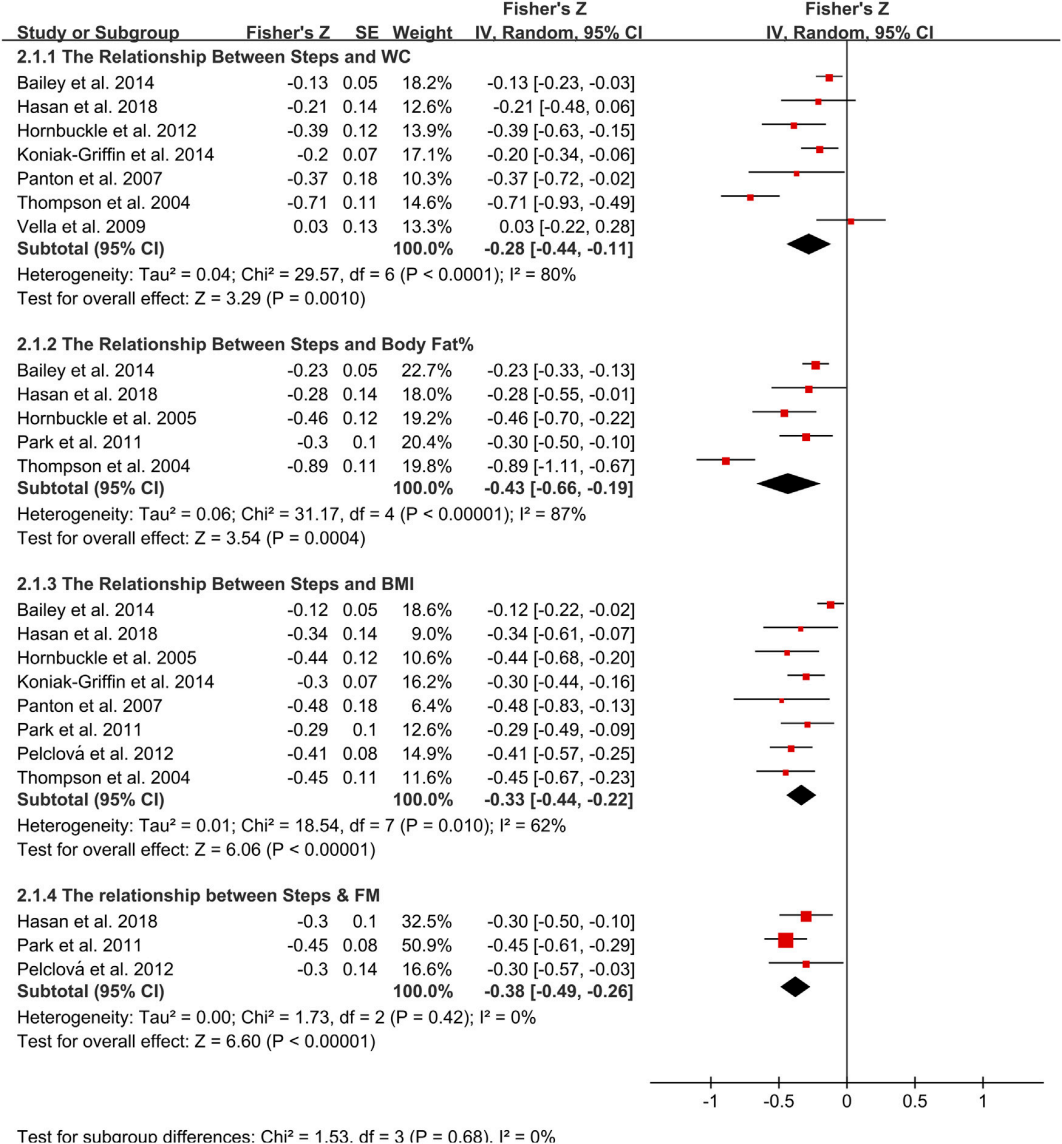
Forest plot of correlation between steps and adiposity outcomes. Overall pooled correlation for random effects model represented by black diamond. Note: BMI, body mass index; FM, fat mass; WC, waist circumference.

15 observational studies and 1 intervention study (Hasan et al., 2018) were included in the correlational meta-analysis (Figure 2). The pooled analysis revealed that daily steps had moderate associations with BMI (*r* = −0.32; 95% CI: −0.44, −0.22; *p* < 0.001; Figure 2), %BF (*r* = −0.41; 95% CI: −0.66, −0.19; *p* < 0.001; Figure 2), and FM (*r* = −0.36; 95% CI: −0.49, −0.26; *p* < 0.001; Figure 2). The between-study heterogeneities were moderate for BMI (I^2^ = 62%, *p* = 0.01), high for %BF (I^2^ = 87%, *p* < 0.001), however, no heterogeneity was shown for FM (I^2^ = 0%, *p* = 0.42). Furthermore, there was a significant but mild association between daily steps and WC (*r* = −0.27; 95% CI: −0.44, −0.11; *p* = 0.001; Figure 2), with a high heterogeneity (I^2^ = 80%, *p* < 0.001). The subgroup analysis revealed that daily steps were significantly associated with BMI and WC in older, but not younger females, with no heterogeneity for BMI (I^2^ = 0%, *p* = 0.68) and high heterogeneity for WC (I^2^ = 81%, *p* = 0.001).. Furthermore, overweight/obese women showed stronger relationships between steps with BMI, %BF, and WC, with reduced heterogeneity for BMI (I^2^ = 0%, *p* = 0.78), %BF (I^2^ = 85%, *p* = 0.001) and WC (I^2^ = 76%, *p* = 0.003). Age and obesity differences were not shown in FM. The magnitude of association between steps with BMI, %BF and WC varied between race, with the strongest associations noted for African American women and weakest in Caucasian women.

**FIGURE 3:**
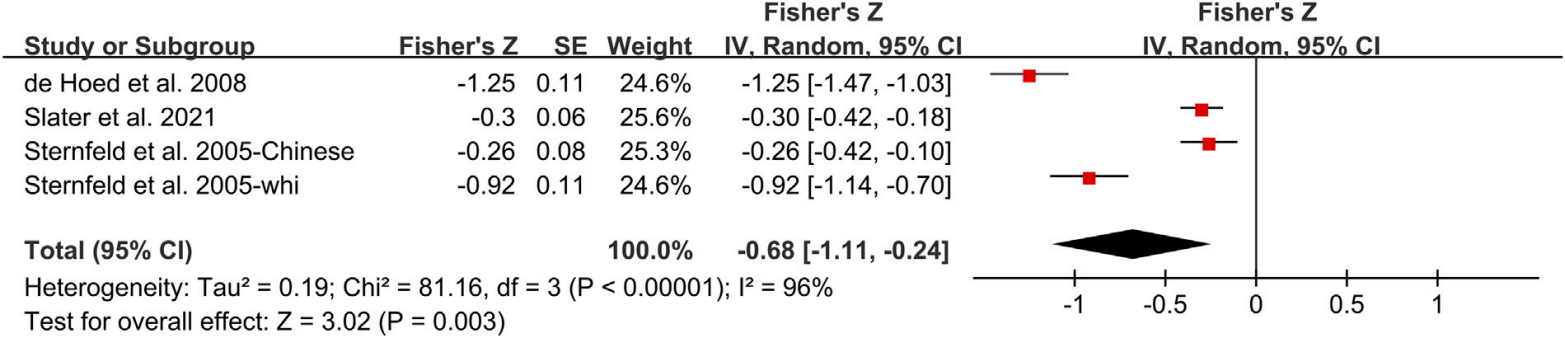
Forest plot of correlation between TPA and %BF. Overall pooled correlation for random effects model represented by black diamond. Note: TPA, total physical activity, %BF, percentage body fat.

There was a more robust and favorable correlation between TPA and %BF (*r* = −0.59; 95% CI: −1.11, −0.24; *p* = 0.003, *n* = 4; Figure 3), with a high heterogeneity of 90%. Subgroup analysis demonstrated that TPA was significantly associated with %BF in Caucasian women, but not Pacific or Chinese women, with a high heterogeneity (I^2^ = 91%, *p* < 0.001). The high heterogeneity between studies could be explained by subgroup analysis according to ethnicity.

**FIGURE 4:**
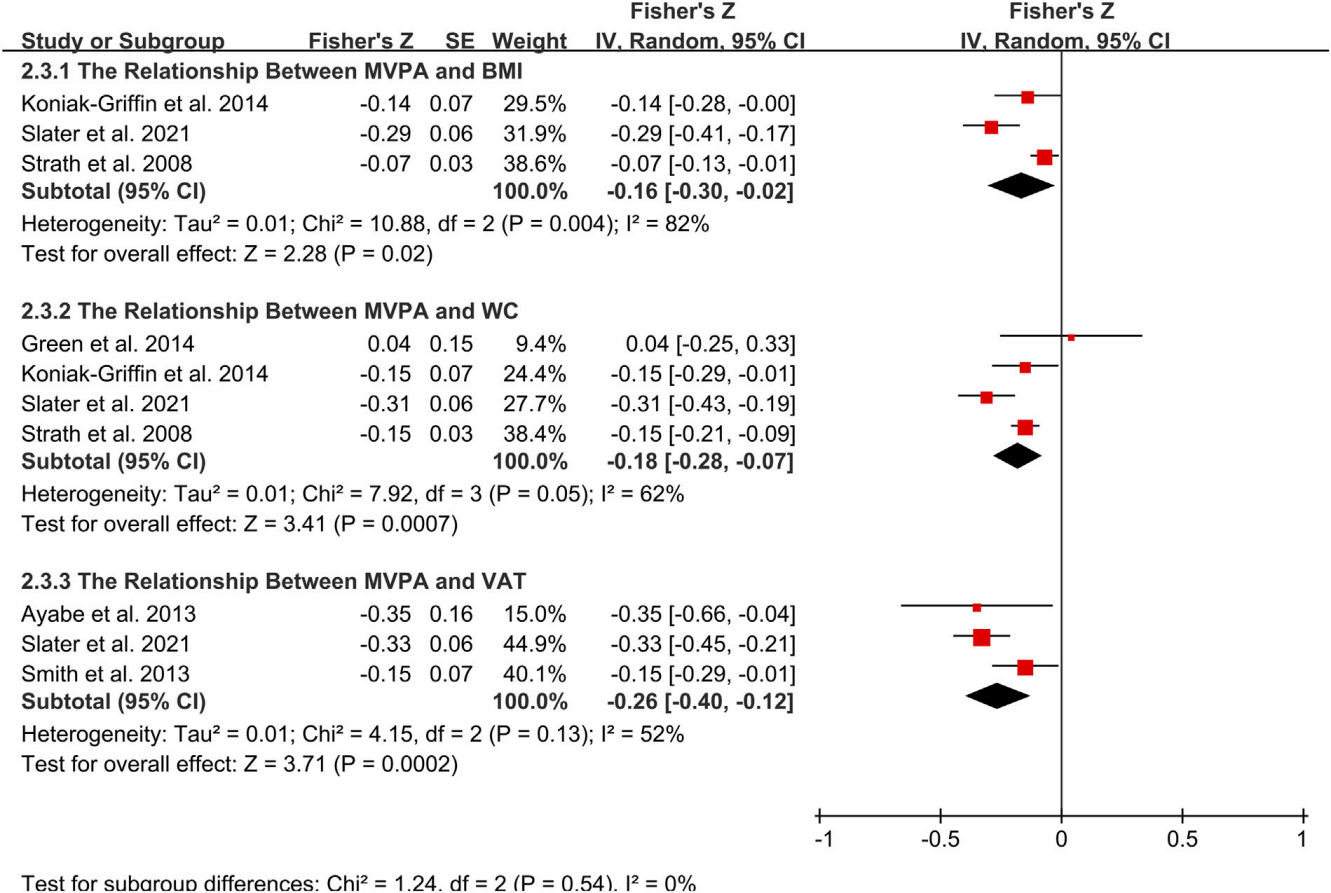
Forest plot of correlation between MVPA and adiposity outcomes. Overall pooled correlation for random effects model represented by black diamond. Note: BMI, body mass index; MVPA, moderate to vigorous physical activity; VAT, visceral adiposity tissue; WC, waist circumference.

Correlational meta-analysis was possible for studies assessing the association between minutes in MVPA with BMI, WC and VAT (Figure 4). There was a significant but weak correlation between minutes in MVPA and BMI (*r* = −0.16; 95% CI: −0.30, −0.02; *p* = 0.02; Figure 2). However, the heterogeneity was shown to be high (I^2^ = 82%, *p* = 0.004). Likewise, MVPA was significantly associated with VAT (*r* = −0.25; 95% CI: −0.4, −0.12; *p* < 0.001) and WC (*r* = −0.18; 95% CI: −0.28, −0.07; *p* < 0.001), with moderate but not significant between-study heterogeneity for VAT (I^2^ = 52%, *p* = 0.13) and for WC (I^2^ = 62%, *p* = 0.05). After performing subgroup analysis, age was the potential source of heterogeneity.

**FIGURE 5:**
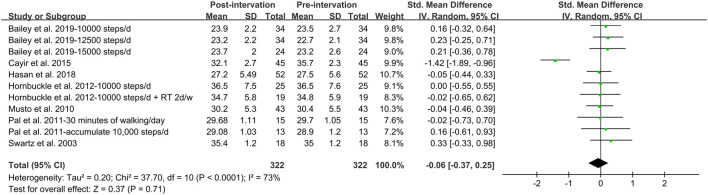
Forest plot of the difference between post- and pre- walking intervention on body mass index. Overall pooled effect for random effects model represented by black diamond.

**FIGURE 6:**
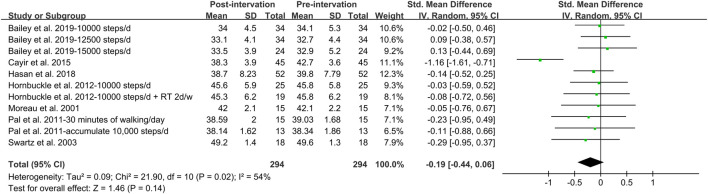
Forest plot of the difference between post- and pre- walking intervention on body fat%. Overall pooled effect for random effects model represented by black diamond.

**FIGURE 7:**
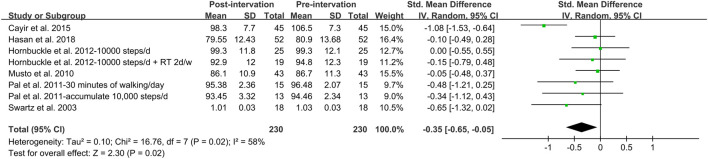
Forest plot of the difference between post- and pre- walking intervention on waist circumference. Overall pooled effect for random effects model represented by black diamond.

**FIGURE 8:**
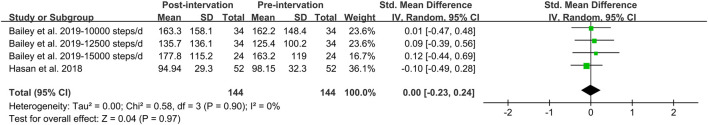
Forest plot of the difference between post- and pre- walking intervention on visceral adiposity tissue. Overall pooled effect for random effects model represented by black diamond.

In addition, meta-analysis was possible for intervention studies investigating the effect of walking intervention on BMI, %BF, VAT and WC (Figures 5, 6, 7, 8). Overall, the walking program resulting in an increase in daily steps had a significant reduction in WC (SMD = −0.35; 95% CI: −0.65, −0.05; *p* = 0.02), with a significant and moderate heterogeneity (I^2^ = 58%, *p* = 0.02). This heterogeneity was driven by the inclusion of women with extremely large mean WC of 106.5 cm (Cayir et al., 2015). Excluding this study resulted homogeneous (I^2^ = 0%) in remaining studies. However, the pooled effect on WC was not significant. Furthermore, there was no significant pooled effect on BMI, %BF or VAT. Subgroup analysis showed that walking intervention had a significant effect on WC in middle-aged women but not in young women, with a moderate heterogeneity (I^2^ = 60%, *p* = 0.02). Subgroup analysis based on age, obesity, menstrual status, country, and ethnicity did not modify the effects of walking protocols on other adiposity indicators.

None of the pooled effects was altered by the subsequent sensitivity analysis.

## 4 Discussion

This systematic review and meta-analysis synthesized studies that investigated the association between objectively measured PA and adiposity in adult women. This was the first study to qualitatively and quantitively synthesize the evidence from both observational and interventional studies.

### 4.1 Association between steps and adiposity outcomes

Meta-analytical evidence (*n* = 15 cross-sectional, *n* = 1 interventional) indicated the favorable association between daily steps and adiposity outcomes such as BMI, %BF, FM, and WC. It is worth noting that, meta-analysis of intervention studies suggested that walking programs were effective in reducing the majority of adiposity indicators including BMI, %BF, and VAT in adult women, but the improvements were not statistically significant. However, a systematic review synthesized the effects of walking on adiposity from RCTs and reported improved outcomes for %BF, WC, and BMI (Murtagh et al., 2015). Likewise, Gao et al. (2016) pooled effects of walking on body composition from 8 RCTs and reported significantly lower BMI, %BF and WC after intervention.

The insignificant effects of walking interventions shown in our meta-analytical findings might be explained by age. According to our subgroup analysis based on the age range, there was difference regarding the effect of walking program on WC. The effect on WC was significant in middle-aged women but not in young women. Likewise, the association between steps with BMI and WC was only significant in middle-aged women. However, this result must be interpreted with caution because the small number of studies represented the young subgroup. Despite this limitation, other studies offered some support that age was associated with the effects of walking program. A systematic review assessed the association of pedometer use with PA and health. The authors reported that the use of pedometers had benefits on increasing steps and participants with older age significantly reduced BMI from baseline (Bravata et al., 2007). Moreover, the positive pooled effect of walking intervention on adiposity was based on mostly middle-aged women in the study by Murtagh et al. (2015) and was concluded by both perimenopausal and postmenopausal women in the study by Gao et al. (2016). It seems that the number of steps required to improve adiposity was not the same for young and middle-aged women. Accumulating 10,000 steps per day was recommended for middle-aged women (Thompson et al., 2004), while 12,000 daily steps was associated with a healthy BMI in young women (Tudor-Lock et al., 2008). The decrease in recommended daily steps was probably due to the decline in energy intake with age.

However, evidence from our intervention study demonstrated that a step recommendation of 12,000 was not effective in weight control among young women. In a study of female university students, there was no significant difference in weight or fat gain among three groups with recommendations of 10,000, 12,500, and 15,000 steps per day respectively (Bailey et al., 2019). This was supported by another intervention study. In an 8-weeks walking program with a target of 12,000 steps per day conducted in young adult, no improvement in BMI was observed at post-measurement (Chiang et al., 2019). Likewise, 10,000 steps per day was ineffective on the reduction of BMI or %BF for middle-aged women (Pal et al., 2011; Swartz et al., 2003; Moreau et al., 2001; Hornbuckle et al., 2012). One of the potential explanations was the walking intensity. The walking program with pedometer was not able to assess the intensity of walking and therefore, slow walking and brisk walking were counted the same by pedometer during the intervention. The increased daily steps seemed to be accumulated from light intensity walking, which was not sufficient to induce a deficit energy balance resulting in weight loss (Bailey et al., 2019). Therefore, in addition to the age-specified recommendation for daily steps, walking intensity also needs consideration when prescribing PA in terms of walking.

### 4.2 Association between total volume of physical activity and adiposity

The meta-analytical result suggested that the total volume of PA was significantly associated with %BF with a moderate effect size. It was consistent with findings from cross-sectional studies (de Hoed and Westerter, 2008; Slater et al., 2021; Tucker and Peterson, 2003). Likewise, a favorable association between TPA and abdominal fat was reported in a cross-sectional study (Diniz et al., 2015). This was supported by a cohort study with a 20-months follow-up, in which authors reported a reduced risk of increasing abdominal fat among women who increased TPA (Davidson et al., 2010). Additionally, Smith et al. (2013) investigated the relationship between accelerometer-derived PA and regional adiposity and found that the amount of TPA was inversely associated with VAT in women.

TPA was defined by the total accelerometer counts in the studies included and it appeared to play an important role in the association with adiposity. de Hoed and Westerter, 2008 reported an additional significant 4% for TPA to explain the variation in %BF when the model was already controlled for age, BMI, and gender. In a cross-sectional study conducted by Tucker and Peterson, 2003, the duration and intensity of PA were both associated with %BF. However, the association was weakened when controlling for TPA. Likewise, another cross-sectional study found that the relationship between higher intensity PA and %BF was negated when adjusting for TPA (Bailey et al., 2015). This could be attributed to the fact that TPA was a cumulative measure insusceptible to the unstandardized cut-points and were able to capture all movements above zero (Van Dyck., 2015). Therefore, TPA provided a lower level of error when investigating the association with adiposity outcomes.

Furthermore, we observed that the association between TPA and adiposity measures was influenced by ethnicity. Findings from subgroup analysis indicated that Caucasian women were more likely to improve their %BF by increasing TPA. In was consistent with other cross-sectional results. In the study by Slater et al. (2021), TPA was favorable associated with %BF in Caucasian women, however, such associations were not significant in Pacific women. Similarly, Sternfeld et al. (2005) reported that TPA was inversely related to %BF and WC only in White subjects, but not in Chinese populations. Results from Ayabe et al. (2013) supported this ethnicity-related difference, at least in part, that TPA was not associated with VAT in Japanese women. The insignificant association observed might be due to the low level of TPA. Although few research investigating ethnicity-specified relationship between TPA and adiposity, numerous previous studies supported our results of Caucasian women, reporting favorable associations between TPA and adiposity outcomes (Wolff-Hughes et al., 2015; Guo et al., 2015; Wanner et al., 2017). Further research is required to explore the impact of ethnicity on the relationship between TPA and adiposity.

### 4.3 Association between physical activity intensities and adiposity

There was consistent evidence that the increased intensity of PA had favorable effects on adiposity outcomes. In an RCTs conducted by Sugawara et al. (2006), vigorous intensity aerobic exercises were evidenced to be more effective than moderate intensity exercises on lowering BMI in postmenopausal women. Evidence from the longitudinal study also corroborated the findings. Bailey et al. (2007) performed a prospective study to investigate the extent to which intensity of PA was associated with changes in %BF. The author also reported a cross-sectional finding that women in a VPA group had lower BF% than those in groups participating in MPA and LPA. Furthermore, women who increased the intensity of PA at follow-ups had reduced risk of fat gains over time. Cross-sectional evidence also supported that %BF was strongly and negatively correlated with the intensity of PA (Tucker and Peterson, 2003; Bailey et al., 2015).

The meta-analysis showed a significant association between MVPA with BMI, WC, and VAT, with a slightly potent association to be shown with fat indicators such as VAT and WC. Hamer et al. (2013) provided longitudinal evidence on the more significant association with WC than with BMI. Furthermore, cross-sectional findings from the present review demonstrated that MVPA had inconsistent effects on BMI and %BF. Women participated in more MVPA did not have a significantly lower BMI but had a more favorable %BF (Diniz et al., 2015; Koniak-Griffin et al., 2014). This was supported by experimental evidence that participating in moderate intensity exercises was able to reduce VAT without weight loss (Lee et al., 2005; van der Heijden et al., 2010). Although BMI was generally used to measure overall adiposity, it provided limited information about the variability of body fat, which appeared to be more important in predicting health, especially reginal fat such as WC and VAT. Despite equivocal evidence for BMI, the favorable effect of MVPA on VAT and WC offered some support for the conclusion that MVPA benefits adiposity.

However, cross-sectional evidence from the present review consistently suggested that there was no relationship between LPA and adiposity (Bailey et al., 2015; Green et al., 2014; Smith et al., 2013). This was supported by a previous review, in which little evidence was reported for the role of LPA to improve body composition (Batacan et al., 2015). Longitudinal evidence from a study examined the association between PA with BMI and WC over 10 years demonstrated that LPA was not associated with changes in BMI or WC (Hamer et al., 2013). Although there was emerging evidence that LPA had benefits for health (Migueles et al., 2021; Ballin et al., 2021), these findings were based on elderly. Our results did not find any relationship between LPA and adiposity outcomes in young women, and the possible health promotion mechanism of LPA need further investigation.

Compared to LPA, VPA had been more consistently shown to be beneficial for all adiposity indicators measured in this review (Ayabe et al., 2013; Sternfeld et al., 2005; Park et al., 2011). This finding again supported the notion that PA intensity was favorably associated with adiposity. Additionally, VPA played a critical role in maintaining weight and preventing weight regain (Phelan et al., 2007). This relationship was corroborated in a cohort study that examined PA and 4-years changes in body mass in 52,498 non-obese people. The study reported that only VPA was effective for weight control among young adults (Byambasukh et al., 2021), suggesting again that MVPA was not associated with better WC amongst young women. Further research is needed to investigate whether age influenced the relationship between PA intensity and obesity.

### 4.4 Association between physical activity duration and adiposity

The evidence from qualitative synthesis in the current review regarding to the association between PA in bout and adiposity was inconsistent. A cross-sectional study examined MVPA accumulated in bouts and non-bouts and reported that MVPA in bouts was significantly associated with the reduction of BMI and WC, while such association was not significant in MVPA non-bout (Strath et al., 2008). It was supported by a cohort study conducted by White et al. (2015), in which the incidence of adiposity was significantly associated with MVPA in 10-min bouts rather than short bouts of MVPA lasting less than 10 min. However, two cross-sectional studies reported opposite findings that the MVPA bouts lasting at least 10 min was not associated with better BMI or WC (Koniak-Griffin et al., 2014; Green et al., 2014). Nonetheless, the favorable association between PA in bouts and adiposity was well documented by previous studies (Pate et al., 1995; Shiroma et al., 2019). The absence of the positive effect of bouts PA might be partially because women often engaged in short bouts of PA, which was normally less than 10 min (Green et al., 2014). This was supported by a previous study, in which nearly two-thirds of MVPA were accumulated by bouts lasting less than 10 min (Cameron et al., 2017). Moreover, the authors indicated that PA in non-bouts was more strongly associated with adiposity than long-sustained PA.

Recent studies tended to offer some support to the notion that every MVPA minute counts. Jefferis et al. (2016) found that there was no difference between MVPA lasting less than 10 min and at least 10 min. Similar findings were also reported by Loprinzi and Cardinal (2013). However, the effect of non-bouts MVPA was not found in our review. This could be explained by the inclusion of lower intensity of PA, which resulted an attenuation of overall effect for non-bouts MVPA. In Strath et al. (2008)’s study, the cut-point used for identifying MVPA was 760 counts per minute (cpm), which was much lower than the most recent recommendation of 2020 cpm by Troiano et al., 2008 and 1952 cpm by Freedson et al., 1998. However, for very short bouts of PA lasting less than 1 min, no significant association was reported with abdominal fat distribution (Ayabe et al., 2013).

Collectively, the association between bouts PA and adiposity outcomes was unclear as the sample women in our review engaged in too little bouts of PA. Although the total amount of MVPA was associated with most adiposity indicators, further research is required to determine whether MVPA accumulated by bouts or non-bouts differed in its effect on adiposity.

#### Implications for practice

The current review suggests that higher daily steps is associated with improvement in indicators of adiposity. Therefore, interventions that target the increase PA may improve adiposity. Meanwhile, if the PA promotion intervention was delivered by walking protocols, walking intensity should be emphasized. In order to improve adiposity, moderate to vigorous intensity was required, while vigorous intensity was preferred.

Additionally, the effect of PA on adiposity indicators was not consistent. BMI appeared to be an unreliable marker to exam the effect of PA on obesity, therefore, adiposity indicators such as %BF, FM and VAT should be considered.

#### Strengths, limitations, and future directions

Strengths of the current study include the use of different types of study designs, the inclusion of objectively determined PA, as well as the wide range of adiposity indicators. This review was the first to analyze the evidence qualitatively and quantitatively from all kinds of study designs, and to explore the association between different PA patterns and the comprehensive adiposity indicators in adult women.

It was important to note that there were some limitations. First, the majority evidence synthesized were of very low to low quality. This was mainly due to the non-randomized study design and the concern with inconsistency in the results across different indicators. Although we compared low-quality evidence to those with high-quality in the discussion, additional research with high quality are required to increase the confidence of findings.

Secondly, most studies included were cross-sectional designs, and most of them assessed associations without controlling for potential confounders such as age and dietary intake. Therefore, the causality and dose-response relationships could not be ascertained. Furthermore, the absence of these confounders weakened the association between PA and adiposity, and our results should be interpreted with caution.

Thirdly, the heterogeneity in the different definition of PA categories, including different cut-points of counts, METs, and vertical acceleration peaks, was a potential source of inconsistent findings and led to indirect comparation of PA intensity. Furthermore, the use of different epochs might contribute to overestimations or underestimates in the amount of PA at a particular intensity. For instance, studies using the longer epochs (e.g., 10 min) were more likely to underestimate the higher intensity PA than those using 60 s epochs. Finally, accelerometer-determined PA was unable to quantify fitness activities such as resistance training, yoga, and Tai chi, as well as unable to precisely calculate the energy expenditure of PA. To deal with these limitations, standardized cut-points, shorter epochs, and pattern recognition should be applied.

In addition, findings from subgroup analyses were limited and should be considered preliminary due to the small number of studies included in each category of subgroup.

## 5 Conclusion

Findings from the present systematic review and meta-analysis provide substantial evidence that objectively derived PA in terms of daily steps, TPA and MVPA is favorably association with most adiposity indicators. TPA has a more potent effect on adiposity, however, this association was influenced by ethnicity. There is no association between LPA and adiposity measures and adiposity is more likely to be benefited from PA performed at higher intensity. These findings must be interpreted with caution since most of the evidence is rated as low, and findings are predominantly derived from cross-sectional analysis. Further high-quality intervention studies are still needed to confidently inform PA recommendations on the volume, intensity and duration.

## Data Availability

The original contributions presented in the study are included in the article/Supplementary Material, further inquiries can be directed to the corresponding author.

## References

[B1] Ainsworth B. E. Haskell W. L. Herrmann S. D. Meckes N. Bassett D. R. Jr. Tudor-Locke C. (2011). 2011 compendium of physical activities: A second update of codes and MET values. Med. Sci. Sports Exerc. 43 (8), 1575–1581. 10.1249/MSS.0b013e31821ece12 21681120

[B2] Althoff T. Sosič R. Hicks J. L. King A. C. Delp S. L. Leskovec J. (2017). Large-scale physical activity data reveal worldwide activity inequality. Nature 547 (7663), 336–339. 10.1038/nature23018 28693034 PMC5774986

[B3] Aune D. Norat T. Leitzmann M. Tonstad S. Vatten L. J. (2015). Physical activity and the risk of type 2 diabetes: A systematic review and dose-response meta-analysis. Eur. J. Epidemiol. 30 (7), 529–542. 10.1007/s10654-015-0056-z 26092138

[B4] Ayabe M. Kumahara H. Morimura K. Sakane N. Ishii K. Tanaka H. (2013). Accumulation of short bouts of non-exercise daily physical activity is associated with lower visceral fat in Japanese female adults. Int. J. Sports Med. 34 (1), 62–67. 10.1055/s-0032-1314814 22903316

[B5] Bailey B. W. Bartholomew C. L. Summerhays C. Deru L. Compton S. Tucker L. A. (2019). The impact of step recommendations on body composition and physical activity patterns in college freshman women: A randomized trial. J. Obes. 2019, 4036825. 10.1155/2019/4036825 31885908 PMC6914918

[B6] Bailey B. W. Borup P. LeCheminant J. D. Tucker L. A. Bromley J. (2015). Examining the relationship between physical activity intensity and adiposity in young women. J. Phys. Act. Health 12 (6), 764–769. 10.1123/jpah.2013-0441 25133866

[B7] Bailey B. W. Borup P. Tucker L. LeCheminant J. Allen M. Hebbert W. (2014). Steps measured by pedometry and the relationship to adiposity in college women. J. Phys. Act. Health 11 (6), 1225–1232. 10.1123/jpah.2012-0255 23963619

[B8] Bailey B. W. Tucker L. A. Peterson T. R. LeCheminant J. D. (2007). A prospective study of physical activity intensity and change in adiposity in middle-aged women. Am. J. Health Promot. 21 (6), 492–497. 10.4278/0890-1171-21.6.492 17674635

[B9] Ballin M. Nordström P. Niklasson J. Nordström A. (2021). Associations of objectively measured physical activity and sedentary time with the risk of stroke, myocardial infarction or all-cause mortality in 70-year-old men and women: A prospective cohort study. Sports Med. 51 (2), 339–349. 10.1007/s40279-020-01356-y 33063268 PMC7846506

[B10] Barker A. R. Gracia-Marco L. Ruiz J. R. Castillo M. J. Aparicio-Ugarriza R. González-Gross M. (2018). Physical activity, sedentary time, TV viewing, physical fitness and cardiovascular disease risk in adolescents: The HELENA study. Int. J. Cardiol. 254, 303–309. 10.1016/j.ijcard.2017.11.080 29221862

[B11] Batacan R. B. Jr. Duncan M. J. Dalbo V. J. Tucker P. S. Fenning A. S. (2015). Effects of light intensity activity on cvd risk factors: A systematic review of intervention studies. Biomed. Res. Int. 2015, 596367. 10.1155/2015/596367 26543862 PMC4620294

[B12] Baudrand R. Domínguez J. M. Tabilo C. Figueroa D. Jimenez M. Eugenin C. (2013). The estimation of visceral adipose tissue with a body composition monitor predicts the metabolic syndrome. J. Hum. Nutr. Diet. 26 (1), 154–158. 10.1111/jhn.12089 23634931

[B13] Bravata D. M. Smith-Spangler C. Sundaram V. Gienger A. L. Lin N. Lewis R. (2007). Using pedometers to increase physical activity and improve health: A systematic review. Jama 298 (19), 2296–2304. 10.1001/jama.298.19.2296 18029834

[B14] Byambasukh O. Vinke P. Kromhout D. Navis G. Corpeleijn E. (2021). Physical activity and 4-year changes in body weight in 52, 498 non-obese people: The lifelines cohort. Int. J. Behav. Nutr. Phys. Act. 18 (1), 75. 10.1186/s12966-021-01141-8 34098972 PMC8186174

[B15] Cameron N. Godino J. Nichols J. F. Wing D. Hill L. Patrick K. (2017). Associations between physical activity and BMI, body fatness, and visceral adiposity in overweight or obese Latino and non-Latino adults. Int. J. Obes. 41 (6), 873–877. 10.1038/ijo.2017.49 PMC5461184 28220040

[B16] Carson V. Ridgers N. D. Howard B. J. Winkler E. A. Healy G. N. Owen N. (2013). Light-intensity physical activity and cardiometabolic biomarkers in US adolescents. PLoS One 8 (8), e71417. 10.1371/journal.pone.0071417 23951157 PMC3739773

[B17] Cayir Y. Aslan S. M. Akturk Z. (2015). The effect of pedometer use on physical activity and body weight in obese women. Eur. J. Sport Sci. 15 (4), 351–356. 10.1080/17461391.2014.940558 25068676

[B18] Chiang T. L. Chen C. Hsu C. H. Lin Y. C. Wu H. J. (2019). Is the goal of 12, 000 steps per day sufficient for improving body composition and metabolic syndrome? The necessity of combining exercise intensity: A randomized controlled trial. BMC Public Health 19 (1), 1215. 10.1186/s12889-019-7554-y 31481039 PMC6724241

[B19] Cohen J. (1988). Statistical power analysis for the behavioral sciences. Hillsdale, N.J: L. Erlbaum Associates.

[B20] Collaboration N. R. F. (2017). Worldwide trends in body-mass index, underweight, overweight, and obesity from 1975 to 2016: A pooled analysis of 2416 population-based measurement studies in 128·9 million children, adolescents, and adults. Lancet 390 (10113), 2627–2642. 10.1016/s0140-6736(17)32129-3 29029897 PMC5735219

[B21] Cooper A. J. Gupta S. R. Moustafa A. F. Chao A. M. (2021). Sex/gender differences in obesity prevalence, comorbidities, and treatment. Curr. Obes. Rep. 10 (4), 458–466. 10.1007/s13679-021-00453-x 34599745

[B22] Davidson L. E. Tucker L. Peterson T. (2010). Physical activity changes predict abdominal fat change in midlife women. J. Phys. Act. Health 7 (3), 316–322. 10.1123/jpah.7.3.316 20551487

[B23] den Hoed M. Westerterp K. R. (2008). Body composition is associated with physical activity in daily life as measured using a triaxial accelerometer in both men and women. Int. J. Obes. 32 (8), 1264–1270. 10.1038/ijo.2008.72 18504444

[B24] Diniz T. A. Fortaleza A. C. Buonani C. Rossi F. E. Neves L. M. Lira F. S. (2015). Relationship between moderate-to-vigorous physical activity, abdominal fat and immunometabolic markers in postmenopausal women. Eur. J. Obstet. Gynecol. Reprod. Biol. 194, 178–182. 10.1016/j.ejogrb.2015.09.013 26412352

[B25] Donnelly J. E. Smith B. K. (2005). Is exercise effective for weight loss with ad libitum diet? Energy balance, compensation, and gender differences. Exerc. Sport Sci. Rev. 33 (4), 169–174. 10.1097/00003677-200510000-00004 16239833

[B26] Dwivedi A. K. Dubey P. Cistola D. P. Reddy S. Y. (2020). Association between obesity and cardiovascular outcomes: Updated evidence from meta-analysis studies. Curr. Cardiol. Rep. 22 (4), 25. 10.1007/s11886-020-1273-y 32166448 PMC12285736

[B27] Egger M. Davey Smith G. Schneider M. Minder C. (1997). Bias in meta-analysis detected by a simple, graphical test. Bmj 315 (7109), 629–634. 10.1136/bmj.315.7109.629 9310563 PMC2127453

[B28] Foright R. M. Presby D. M. Sherk V. D. Kahn D. Checkley L. A. Giles E. D. (2018). Is regular exercise an effective strategy for weight loss maintenance? Physiol. Behav. 188, 86–93. 10.1016/j.physbeh.2018.01.025 29382563 PMC5929468

[B29] Freedson P. S. Melanson E. Sirard J. (1998). Calibration of the computer science and applications, Inc. accelerometer. Med. Sci. Sports Exerc. 30 (5), 777–781. 10.1097/00005768-199805000-00021 9588623

[B30] Gaesser G. A. Angadi S. S. (2021). Obesity treatment: Weight loss versus increasing fitness and physical activity for reducing health risks. iScience 24 (10), 102995. 10.1016/j.isci.2021.102995 34755078 PMC8560549

[B31] Gao H. L. Gao H. X. Sun F. M. Zhang L. (2016). Effects of walking on body composition in perimenopausal and postmenopausal women: A systematic review and meta-analysis. Menopause 23 (8), 928–934. 10.1097/gme.0000000000000627 27187009

[B32] Graff S. K. Alves B. C. Toscani M. K. Spritzer P. M. (2012). Benefits of pedometer-measured habitual physical activity in healthy women. Appl. Physiol. Nutr. Metab. 37 (1), 149–156. 10.1139/h11-145 22288927

[B33] Green A. N. McGrath R. Martinez V. Taylor K. Paul D. R. Vella C. A. (2014). Associations of objectively measured sedentary behavior, light activity, and markers of cardiometabolic health in young women. Eur. J. Appl. Physiol. 114 (5), 907–919. 10.1007/s00421-014-2822-0 24463602

[B34] Guo W. Bradbury K. E. Reeves G. K. Key T. J. (2015). Physical activity in relation to body size and composition in women in UK Biobank. Ann. Epidemiol. 25 (6), 406–413. e406. 10.1016/j.annepidem.2015.01.015 25749558

[B35] Guthold R. Stevens G. A. Riley L. M. Bull F. C. (2018). Worldwide trends in insufficient physical activity from 2001 to 2016: A pooled analysis of 358 population-based surveys with 1·9 million participants. Lancet. Glob. Health 6 (10), e1077–e1086. 10.1016/s2214-109x(18)30357-7 30193830

[B36] Guyatt G. Oxman A. D. Akl E. A. Kunz R. Vist G. Brozek J. (2011). GRADE guidelines: 1. Introduction-GRADE evidence profiles and summary of findings tables. J. Clin. Epidemiol. 64 (4), 383–394. 10.1016/j.jclinepi.2010.04.026 21195583

[B37] Hamer M. Brunner E. J. Bell J. Batty G. D. Shipley M. Akbaraly T. (2013). Physical activity patterns over 10 years in relation to body mass index and waist circumference: The whitehall II cohort study. Obes. (Silver Spring) 21 (12), E755–E761. 10.1002/oby.20446 23512753

[B38] Hasan H. Attlee A. Jan Bin Jan Mohamed H. Aris N. Bin Wan Muda W. A. M. (2018). Counting footsteps with a pedometer to improve HMW adiponectin and metabolic syndrome among young female adults in the united Arab emirates. J. Obes. 2018, 1597840. 10.1155/2018/1597840 30631594 PMC6304855

[B39] Herman K. M. Hopman W. M. Craig C. L. (2011). Sex differences in the association of youth body mass index to adult health-related quality of life: The physical activity longitudinal study. Can. J. Public Health 102 (1), 42–46. 10.1007/bf03404875 21485965 PMC6974263

[B40] Higgins J. P. Altman D. G. Gøtzsche P. C. Jüni P. Moher D. Oxman A. D. (2011). The Cochrane Collaboration's tool for assessing risk of bias in randomised trials. Bmj 343, d5928. 10.1136/bmj.d5928 22008217 PMC3196245

[B41] Higgins J. P. T. Thompson S. G. Deeks J. J. Altman D. G. (2003). Measuring inconsistency in meta-analyses. BMJ 327 (7414), 557–560. 10.1136/bmj.327.7414.557 12958120 PMC192859

[B42] Holliday A. Burgin A. Fernandez E. V. Fenton S. A. M. Thielecke F. Blannin A. K. (2018). Points-based physical activity: A novel approach to facilitate changes in body composition in inactive women with overweight and obesity. BMC Public Health 18 (1), 261. 10.1186/s12889-018-5125-2 29454318 PMC5816513

[B43] Hornbuckle L. M. Bassett D. R. Jr. Thompson D. L. (2005). Pedometer-determined walking and body composition variables in African-American women. Med. Sci. Sports Exerc. 37 (6), S305. 10.1249/00005768-200505001-01591 15947735

[B44] Hornbuckle L. M. Liu P. Y. Ilich J. Z. Kim J. S. Arjmandi B. H. Panton L. B. (2012). Effects of resistance training and walking on cardiovascular disease risk in African-American women. Med. Sci. Sports Exerc. 44 (3), 525–533. 10.1249/MSS.0b013e31822e5a12 21778912

[B45] Jakicic J. M. Davis K. K. (2011). Obesity and physical activity. Psychiatr. Clin. North Am. 34 (4), 829–840. 10.1016/j.psc.2011.08.009 22098807

[B46] Jefferis B. J. Parsons T. J. Sartini C. Ash S. Lennon L. T. Wannamethee S. G. (2016). Does duration of physical activity bouts matter for adiposity and metabolic syndrome? A cross-sectional study of older British men. Int. J. Behav. Nutr. Phys. Act. 13 (1), 36. 10.1186/s12966-016-0361-2 26980183 PMC4793648

[B47] Johnson N. A. Sultana R. N. Brown W. J. Bauman A. E. Gill T. (2021). Physical activity in the management of obesity in adults: A position statement from exercise and sport science Australia. J. Sci. Med. Sport 24 (12), 1245–1254. 10.1016/j.jsams.2021.07.009 34531124

[B48] Julian V. Ciba I. Olsson R. Dahlbom M. Furthner D. Gomahr J. (2022). Association between metabolic syndrome diagnosis and the physical activity—sedentary profile of adolescents with obesity: A complementary analysis of the beta-judo study. Nutrients 14 (1), 60. 10.3390/nu14010060 PMC8746544 35010936

[B49] Kahn H. S. Bullard K. M. Barker L. E. Imperatore G. (2012). Differences between adiposity indicators for predicting all-cause mortality in a representative sample of United States non-elderly adults. PLoS One 7 (11), e50428. 10.1371/journal.pone.0050428 23226283 PMC3511554

[B50] Kapoor N. Lotfaliany M. Sathish T. Thankappan K. R. Thomas N. Furler J. (2020). Obesity indicators that best predict type 2 diabetes in an Indian population: Insights from the Kerala diabetes prevention program. J. Nutr. Sci. 9, e15. 10.1017/jns.2020.8 32328239 PMC7163399

[B51] Kelly T. Yang W. Chen C. S. Reynolds K. He J. (2008). Global burden of obesity in 2005 and projections to 2030. Int. J. Obes. 32 (9), 1431–1437. 10.1038/ijo.2008.102 18607383

[B52] Kim S. Singh H. (2022). Sex-specific associations among total bone-specific physical activity score, aortic parameters, and body composition in healthy young adults. J. Exerc. Sci. Fit. 20 (1), 27–31. 10.1016/j.jesf.2021.12.002 34976077 PMC8683586

[B53] Koniak-Griffin D. Brecht M. L. Takayanagi S. Villegas J. Melendrez M. (2014). Physical activity and cardiometabolic characteristics in overweight Latina women. J. Immigr. Minor. Health 16 (5), 856–864. 10.1007/s10903-013-9782-z 23355122 PMC3758377

[B54] Kossi O. Lacroix J. Ferry B. Batcho C. S. Julien-Vergonjanne A. Mandigout S. (2021). Reliability of ActiGraph GT3X+ placement location in the estimation of energy expenditure during moderate and high-intensity physical activities in young and older adults. J. Sports Sci. 39 (13), 1489–1496. 10.1080/02640414.2021.1880689 33514289

[B55] Lee S. Kuk J. L. Davidson L. E. Hudson R. Kilpatrick K. Graham T. E. (2005). Exercise without weight loss is an effective strategy for obesity reduction in obese individuals with and without Type 2 diabetes. J. Appl. Physiol. 99 (3), 1220–1225. 10.1152/japplphysiol.00053.2005 15860689

[B56] Loprinzi P. D. Cardinal B. J. (2013). Association between biologic outcomes and objectively measured physical activity accumulated in ≥ 10-minute bouts and <10-minute bouts. Am. J. Health Promot. 27 (3), 143–151. 10.4278/ajhp.110916-QUAN-348 23286590

[B57] Loprinzi P. D. (2017). Light-intensity physical activity and all-cause mortality. Am. J. Health Promot. 31 (4), 340–342. 10.4278/ajhp.150515-ARB-882 26730555

[B58] Marschollek M. (2015). Physical activity event regularity and health outcome - 'Undiscovered country' in cohort accelerometer data. Stud. Health Technol. Inf. 210, 657–659. 25991230

[B59] Migueles J. H. Lee I. M. Sanchez C. C. Ortega F. B. Buring J. E. Shiroma E. J. (2021). Revisiting the association of sedentary behavior and physical activity with all-cause mortality using a compositional approach: The women's health study. Int. J. Behav. Nutr. Phys. Act. 18 (1), 104. 10.1186/s12966-021-01173-0 34376213 PMC8353824

[B60] Moher D. Liberati A. Tetzlaff J. Altman D. G. (2009). Preferred reporting items for systematic reviews and meta-analyses: The PRISMA statement. PLoS Med. 6 (7), e1000097. 10.1371/journal.pmed.1000097 19621072 PMC2707599

[B61] Moreau K. L. Degarmo R. Langley J. McMahon C. Howley E. T. Bassett D. R. Jr. (2001). Increasing daily walking lowers blood pressure in postmenopausal women. Med. Sci. Sports Exerc. 33 (11), 1825–1831. 10.1097/00005768-200111000-00005 11689731

[B62] Murray C. B. Patel K. V. Twiddy H. Sturgeon J. A. Palermo T. M. (2021). Age differences in cognitive-affective processes in adults with chronic pain. Eur. J. Pain 25 (5), 1041–1052. 10.1002/ejp.1725 33405280 PMC8055045

[B63] Murtagh E. M. Nichols L. Mohammed M. A. Holder R. Nevill A. M. Murphy M. H. (2015). The effect of walking on risk factors for cardiovascular disease: An updated systematic review and meta-analysis of randomised control trials. Prev. Med. 72, 34–43. 10.1016/j.ypmed.2014.12.041 25579505

[B64] Musto A. Jacobs K. Nash M. DelRossi G. Perry A. (2010). The effects of an incremental approach to 10, 000 steps/day on metabolic syndrome components in sedentary overweight women. J. Phys. Act. Health 7 (6), 737–745. 10.1123/jpah.7.6.737 21088304

[B65] Oliveros E. Somers V. K. Sochor O. Goel K. Lopez-Jimenez F. (2014). The concept of normal weight obesity. Prog. Cardiovasc. Dis. 56 (4), 426–433. 10.1016/j.pcad.2013.10.003 24438734

[B66] Pal S. Cheng C. Ho S. (2011). The effect of two different health messages on physical activity levels and health in sedentary overweight, middle-aged women. BMC Public Health 11, 204. 10.1186/1471-2458-11-204 21453540 PMC3078883

[B67] Panton L. B. Kushnick M. R. Kingsley J. D. Moffatt R. J. Haymes E. M. Toole T. (2007). Pedometer measurement of physical activity and chronic disease risk factors of obese lower socioeconomic status African American women. J. Phys. Act. Health 4 (4), 447–458. 18209235

[B68] Park J. Ishikawa-Takata K. Tanaka S. Hikihara Y. Ohkawara K. Watanabe S. (2011). Relation of body composition to daily physical activity in free-living Japanese adult women. Br. J. Nutr. 106 (7), 1117–1127. 10.1017/s0007114511001358 21736836

[B69] Pate R. R. Pratt M. Blair S. N. Haskell W. L. Macera C. A. Bouchard C. (1995). Physical activity and public health. A recommendation from the centers for disease control and prevention and the American college of sports medicine. Jama 273 (5), 402–407. 10.1001/jama.273.5.402 7823386

[B70] Pelclová J. Gába A. Tlučáková L. Pośpiech D. (2012). Association between physical activity (PA) guidelines and body composition variables in middle-aged and older women. Arch. Gerontol. Geriatr. 55 (2), e14–20. 10.1016/j.archger.2012.06.014 22819080

[B71] Peterson R. A. Brown S. P. (2005). On the use of beta coefficients in meta-analysis. J. Appl. Psychol. 90 (1), 175–181. 10.1037/0021-9010.90.1.175 15641898

[B72] Phelan S. Roberts M. Lang W. Wing R. R. (2007). Empirical evaluation of physical activity recommendations for weight control in women. Med. Sci. Sports Exerc. 39 (10), 1832–1836. 10.1249/mss.0b013e31812383c3 17909412 PMC2699680

[B73] Ramakrishnan R. He J. R. Ponsonby A. L. Woodward M. Rahimi K. Blair S. N. (2021). Objectively measured physical activity and all cause mortality: A systematic review and meta-analysis. Prev. Med. 143, 106356. 10.1016/j.ypmed.2020.106356 33301824

[B74] Ramsey K. A. Zhou W. Rojer A. G. M. Reijnierse E. M. Maier A. B. (2022). Associations of objectively measured physical activity and sedentary behaviour with fall-related outcomes in older adults: A systematic review. Ann. Phys. Rehabil. Med. 65 (2), 101571. 10.1016/j.rehab.2021.101571 34530151

[B75] Rosenberg L. Kipping-Ruane K. L. Boggs D. A. Palmer J. R. (2013). Physical activity and the incidence of obesity in young African-American women. Am. J. Prev. Med. 45 (3), 262–268. 10.1016/j.amepre.2013.04.016 23953351 PMC3774527

[B76] Schardt C. Adams M. B. Owens T. Keitz S. Fontelo P. (2007). Utilization of the PICO framework to improve searching PubMed for clinical questions. BMC Med. Inf. Decis. Mak. 7, 16. 10.1186/1472-6947-7-16 PMC1904193 17573961

[B77] Shiroma E. J. Lee I. M. Schepps M. A. Kamada M. Harris T. B. (2019). Physical activity patterns and mortality: The weekend warrior and activity bouts. Med. Sci. Sports Exerc. 51 (1), 35–40. 10.1249/mss.0000000000001762 30138219 PMC6295264

[B78] Sims S. T. Larson J. C. Lamonte M. J. Michael Y. L. Martin L. W. Johnson K. C. (2012). Physical activity and body mass: Changes in younger versus older postmenopausal women. Med. Sci. Sports Exerc. 44 (1), 89–97. 10.1249/MSS.0b013e318227f906 21659897

[B79] Sirard J. R. Pate R. R. (2001). Physical activity assessment in children and adolescents. Sports Med. 31 (6), 439–454. 10.2165/00007256-200131060-00004 11394563

[B80] Slater J. Kruger R. Douwes J. O'Brien W. J. Corbin M. Miles-Chan J. L. (2021). Objectively measured physical activity is associated with body composition and metabolic profiles of pacific and New Zealand European women with different metabolic disease risks. Front. Physiol. 12, 684782. 10.3389/fphys.2021.684782 34122148 PMC8188826

[B81] Smith H. A. Storti K. L. Arena V. C. Kriska A. M. Gabriel K. K. P. Sutton-Tyrrell K. (2013). Associations between accelerometer-derived physical activity and regional adiposity in young men and women. Obesity 21 (6), 1299–1305. 10.1002/oby.20308 23408709 PMC3716839

[B82] Spittaels H. Van Cauwenberghe E. Verbestel V. De Meester F. Van Dyck D. Verloigne M. (2012). Objectively measured sedentary time and physical activity time across the lifespan: A cross-sectional study in four age groups. Int. J. Behav. Nutr. Phys. Act. 9, 149. 10.1186/1479-5868-9-149 23249449 PMC3542099

[B83] Sternfeld B. Bhat A. K. Wang H. Sharp T. Quesenberry C. P. Jr. (2005). Menopause, physical activity, and body composition/fat distribution in midlife women. Med. Sci. Sports Exerc. 37 (7), 1195–1202. 10.1249/01.mss.0000170083.41186.b1 16015138

[B84] Strath S. J. Holleman R. G. Ronis D. L. Swartz A. M. Richardson C. R. (2008). Objective physical activity accumulation in bouts and nonbouts and relation to markers of obesity in US adults. Prev. Chronic Dis. 5 (4), A131. 18793519 PMC2578774

[B85] Sugawara J. Otsuki T. Tanabe T. Hayashi K. Maeda S. Matsuda M. (2006). Physical activity duration, intensity, and arterial stiffening in postmenopausal women. Am. J. Hypertens. 19 (10), 1032–1036. 10.1016/j.amjhyper.2006.03.008 17027823

[B86] Swartz A. M. Strath S. J. Bassett D. R. Moore J. B. Redwine B. A. Groër M. (2003). Increasing daily walking improves glucose tolerance in overweight women. Prev. Med. 37 (4), 356–362. 10.1016/s0091-7435(03)00144-0 14507493

[B87] Thompson D. L. Rakow J. Perdue S. M. (2004). Relationship between accumulated walking and body composition in middle-aged women. Med. Sci. Sports Exerc. 36 (5), 911–914. 10.1249/01.mss.0000126787.14165.b3 15126729

[B88] Tolonen S. Sievänen H. Hirvensalo M. Laaksonen M. Mikkilä V. Pälve K. (2018). Higher step count is associated with greater bone mass and strength in women but not in men. Arch. Osteoporos. 13 (1), 20. 10.1007/s11657-018-0425-9 29511893

[B89] Toth M. J. Tchernof A. Sites C. K. Poehlman E. T. (2000). Effect of menopausal status on body composition and abdominal fat distribution. Int. J. Obes. Relat. Metab. Disord. 24 (2), 226–231. 10.1038/sj.ijo.0801118 10702775

[B90] Troiano R. P. Berrigan D. Dodd K. W. Mâsse L. C. Tilert T. McDowell M. (2008). Physical activity in the United States measured by accelerometer. Med. Sci. Sports Exerc. 40 (1), 181–188. 10.1249/mss.0b013e31815a51b3 18091006

[B91] Tselha N. Shimrah C. Kulshreshtha M. Devi N. K. (2019). Association between hypertension and adiposity indicators: A study among the muslim population of Uttar Pradesh. Diabetes Metab. Syndr. 13 (4), 2335–2338. 10.1016/j.dsx.2019.05.016 31405639

[B92] Tucker L. A. Peterson T. R. (2003). Objectively measured intensity of physical activity and adiposity in middle-aged women. Obes. Res. 11 (12), 1581–1587. 10.1038/oby.2003.210 14694224

[B93] Tudor-Locke C. Bassett D. R. Jr. Rutherford W. J. Ainsworth B. E. Chan C. B. Croteau K. (2008). BMI-referenced cut points for pedometer-determined steps per day in adults. J. Phys. Act. Health 5, S126–S139. 10.1123/jpah.5.s1.s126 18364517 PMC2866423

[B94] Tudor-Locke C. Burton N. W. Brown W. J. (2009). Leisure-time physical activity and occupational sitting: Associations with steps/day and BMI in 54-59 year old Australian women. Prev. Med. 48 (1), 64–68. 10.1016/j.ypmed.2008.10.016 19027786

[B95] van der Heijden G. J. Wang Z. J. Chu Z. D. Sauer P. J. Haymond M. W. Rodriguez L. M. (2010). A 12-week aerobic exercise program reduces hepatic fat accumulation and insulin resistance in obese, Hispanic adolescents. Obes. (Silver Spring) 18 (2), 384–390. 10.1038/oby.2009.274 19696755

[B96] Van Dyck D. Cerin E. De Bourdeaudhuij I. Hinckson E. Reis R. S. Davey R. (2015). International study of objectively measured physical activity and sedentary time with body mass index and obesity: IPEN adult study. Int. J. Obes. 39 (2), 199–207. 10.1038/ijo.2014.115 PMC4282619 24984753

[B97] Vella C. A. Ontiveros D. Zubia R. Y. Dalleck L. (2011). Physical activity recommendations and cardiovascular disease risk factors in young Hispanic women. J. Sports Sci. 29 (1), 37–45. 10.1080/02640414.2010.520727 21086215

[B98] Vella C. A. Zubia R. Y. Ontiveros D. Cruz M. L. (2009). Physical activity, cardiorespiratory fitness, and metabolic syndrome in young Mexican and Mexican-American women. Appl. Physiol. Nutr. Metab. 34 (1), 10–17. 10.1139/h08-134 19234580

[B99] Wagner K. H. Brath H. (2012). A global view on the development of non communicable diseases. Prev. Med. 54 (1), S38–S41. 10.1016/j.ypmed.2011.11.012 22178469

[B100] Wanner M. Richard A. Martin B. Faeh D. Rohrmann S. (2017). Associations between self-reported and objectively measured physical activity, sedentary behavior and overweight/obesity in NHANES 2003-2006. Int. J. Obes. 41 (1), 186–193. 10.1038/ijo.2016.168 27677618

[B101] Wells G. S. B. O’Connell D. Peterson J. Welch V. Losos M. Tugwell P. (2019). The Newcastle-Ottawa Scale (NOS) for assessing the quality of nonrandomised studies in meta-analyses [Online]. Available: http://www.ohri.ca/programs/clinical_epidemiology/oxford.asp (Accessed March 5, 2022).

[B102] White D. K. Gabriel K. P. Kim Y. Lewis C. E. Sternfeld B. (2015). Do short spurts of physical activity benefit cardiovascular health? The CARDIA study. Med. Sci. Sports Exerc. 47 (11), 2353–2358. 10.1249/mss.0000000000000662 25785930 PMC4573767

[B103] Whitlock G. Lewington S. Sherliker P. Clarke R. Emberson J. Halsey J. (2009). Body-mass index and cause-specific mortality in 900 000 adults: Collaborative analyses of 57 prospective studies. Lancet 373 (9669), 1083–1096. 10.1016/s0140-6736(09)60318-4 19299006 PMC2662372

[B104] Wolff-Hughes D. L. Fitzhugh E. C. Bassett D. R. Churilla J. R. (2015). Total activity counts and bouted minutes of moderate-to-vigorous physical activity: Relationships with cardiometabolic biomarkers using 2003-2006 NHANES. J. Phys. Act. Health 12 (5), 694–700. 10.1123/jpah.2013-0463 25109602

