# Supplementary material for: Objectively determined physical activity and adiposity measures in adult women: A systematic review and meta-analysis
Source: Front Physiol. 2022 Aug 23;13:935892. doi: 10.3389/fphys.2022.935892 (PMC9445154; doi:10.3389/fphys.2022.935892)
Supplement: Supplementary file 1 [file Table1.DOCX]

**Supplementary Table A: Search strategies**

| Database |  | PubMed (n=2954) |
| --- | --- | --- |
| Date |  | January 1, 1990-January 31, 2022 |
| Search Terms | #1 | (Accelerometry[Mesh] OR Accelero*[TIAB] OR Actigraphy[MAJR] OR actigra* [TIAB]) Filters: Young Adult: 19-24 years, Adult: 19-44 years, Middle Aged: 45-64 years, Female |
|  | #2 | (objectively[TIAB] AND assessed[TIAB] AND physical[TIAB] AND activity[TIAB]) Filters: Young Adult: 19-24 years, Adult: 19-44 years, Middle Aged: 45-64 years, Female |
|  | #3 | (objectively[TIAB] AND measured[TIAB] AND physical[TIAB] AND activity[TIAB]) Filters: Young Adult: 19-24 years, Adult: 19-44 years, Middle Aged: 45-64 years, Female |
|  | #4 | pedometer[TIAB] Filters: Young Adult: 19-24 years, Adult: 19-44 years, Middle Aged: 45-64 years, Female |
|  | #5 | "Body Composition"[Mesh] OR BMI OR “body mass index” OR fat OR overweight OR obes* OR adipos* OR waist OR “fat mass” OR “fat free mass” OR “body fat” Filters: Young Adult: 19-24 years, Adult: 19-44 years, Middle Aged: 45-64 years, Female |
|  |  | #1 OR #2 OR #3 OR #4 AND #5 |
|  |  | (((((Accelerometry[Mesh] OR Accelero*[TIAB] OR Actigraphy[Mesh] OR actigra* [TIAB]) AND (adult[Filter] OR middleaged[Filter] OR youngadult[Filter])) OR ((objectively[TIAB] AND assessed[TIAB] AND physical[TIAB] AND activity[TIAB]) AND (youngadult[Filter] OR adult[Filter] OR middleaged[Filter]))) OR ((objectively[TIAB] AND measured[TIAB] AND physical[TIAB] AND activity[TIAB]) AND (youngadult[Filter] OR adult[Filter] OR middleaged[Filter]))) OR (pedometer[TIAB] AND (youngadult[Filter] OR adult[Filter] OR middleaged[Filter]))) AND ("Body Composition"[Mesh] OR BMI OR "body mass index" OR fat OR overweight OR obes* OR adipos* OR waist OR "fat mass" OR "fat free mass" OR "body fat" AND ((female[Filter]) AND (english[Filter]) AND (youngadult[Filter] OR adult[Filter] OR middleaged[Filter]))) |
| Database |  | SCOPUS (n=921) |
| Date |  | January 1, 1990-January 31, 2022 |
|  | #1 | TITLE-ABS-KEY ( accelerometry OR accelero* OR actigra* OR actigraphy ) |
|  | #2 | TITLE-ABS-KEY ( ( objectively AND measured AND physical AND activity ) ) |
|  | #3 | TITLE-ABS-KEY ( ( objectively AND assessed AND physical AND activity ) ) |
|  | #4 | TITLE-ABS-KEY ( pedometer ) |
|  | #5 | "Body Composition" OR BMI OR “body mass index” OR fat OR overweight OR obes* OR adipos* OR waist OR “fat mass” OR “fat free mass” OR “body fat” |
|  |  | #1 OR #2 OR #3 OR #4 AND #5 |
|  |  | (TITLE-ABS-KEY(accelerometry OR accelero* OR actigra* OR actigraphy) OR TITLE-ABS-KEY(objectively AND measured AND physical AND activity) OR TITLE-ABS-KEY(objectively AND assessed AND physical AND activity) OR TITLE-ABS-KEY(pedometer)) AND (TITLE-ABS-KEY ("Body Composition" OR bmi OR "body mass index" OR fat OR overweight OR obes* OR adipos* OR waist OR "fat mass" OR "fat free mass" OR "body fat")) AND NOT ((child*) OR (old*) OR (eld*) OR (pregnan*) OR (disable*) OR (athlete)) AND ( LIMIT-TO (DOCTYPE, "ar" ) )  AND  ( LIMIT-TO ( LANGUAGE ,  "English" ) ) |
| Database |  | Web of Science (n=4666) |
| Date |  | January 1, 1990-January 31, 2022 |
|  | #1 | TS=(accelerometry OR accelero* OR actigra* OR actigraphy) |
|  | #2 | TS=(objectively AND measured AND physical AND activity) |
|  | #3 | TS=(objectively AND assessed AND physical AND activity) |
|  | #4 | TS=(pedometer) |
|  | #5 | TS=(“Body Composition” OR BMI OR “body mass index” OR fat OR overweight OR obes* OR adipos* OR waist OR “fat mass” OR “fat free mass” OR “body fat”) |
|  |  | (((((#1) OR #2) OR #3) OR #4) AND #5) NOT ALL=( ((child*) OR (old*) OR (eld*) OR (pregnan*) OR (disable*) OR (athlete))) |
| Database |  | The Cochrane library (n=1636) |
| Date |  | January 1, 1990-January 31, 2022 |
|  | #1 | MeSH descriptor: [Accelerometry] this term only |
|  | #2 | accelero* OR actigra* OR actigraphy |
|  | #3 | #1 OR #2 |
|  | #4 | objectively AND measured AND physical AND activity:ti,ab,kw |
|  | #5 | objectively AND assessed AND physical AND activity:ti,ab,kw |
|  | #6 | pedometer |
|  | #7 | #3 OR #4 OR #5 OR #6 |
|  | #8 | "Body Composition" OR BMI OR “body mass index” OR fat OR overweight OR obes* OR adipos* OR waist OR “fat mass” OR “fat free mass” OR “body fat” |
|  | #9 | MeSH descriptor: [Chronic Disease] this term only |
|  | #10 | #8 OR #9 |
|  | #11 | (child*) OR (old*) OR (eld*) OR (pregnan*) OR (disable*) OR (athlete) |
|  | #12 | #7 AND #10 NOT #11 |

**Supplementary Table B: methodological quality of studies**

**Supplementary Table B.1 Assessment of methodological quality of nonrandomized studies (Newcastle Ottawa Scale).**

| Study | Selection | | | | Comparability | Outcome | | | Total | Quality |
| --- | --- | --- | --- | --- | --- | --- | --- | --- | --- | --- |
|  | Representativeness of the exposed cohort | Selection of non-exposed cohort | Ascertainment of exposure | Outcome not present at start of study | Adjusted for age and other important covariates | Assessment of outcome | Follow-up long enough for outcomes to occur | Adequacy of follow up |  |  |
| Ayabe et al. 2013 | - | * | * | * | ** | * | n/a | n/a | 6 | High |
| Bailey et al. 2015 | - | * | * | * | ** | * | n/a | n/a | 6 | High |
| Bailey et al. 2014 | - | * | * | * | ** | * | n/a | n/a | 6 | High |
| Bailey et al. 2007 | * | * | - | * | ** | * | * | - | 7 | High |
| de Hoed et al. 2008 | - | * | - | * | - | * | n/a | n/a | 3 | Low |
| Diniz et al. 2015 | - | * | * | * | * | * | n/a | n/a | 5 | High |
| Graff et al. 2012 | - | * | - | * | - | * | n/a | n/a | 3 | Low |
| Green et al. 2014 | * | * | * | * | - | * | n/a | n/a | 5 | High |
| Hasan et al. 2018 | - | * | * | * | - | * | - | * | 5 | High |
| Hornbuckle et al. 2005 | * | * | * | * | ** | * | n/a | n/a | 7 | High |
| Koniak-Griffin et al. 2014 | * | * | - | * | - | * | n/a | n/a | 4 | High |
| Musto et al. 2010 | - | * | - | * | - | * | - | * | 4 | Low |
| Panton et al. 2007 | - | * | - | * | - | * | n/a | n/a | 3 | Low |
| Park et al. 2011 | - | * | - | * | - | * | n/a | n/a | 3 | Low |
| Pelclová et al. 2012 | - | * | * | * | - | * | n/a | n/a | 4 | Low |
| Phelan et al. 2007 | - | - | * | * | - | * | n/a | n/a | 3 | Low |
| Slater et al. 2021 | * | * | * | * | ** | * | n/a | n/a | 7 | High |
| Smith et al. 2013 | * | * | * | * | ** | * | n/a | n/a | 7 | High |
| Sternfeld et al. 2005 | * | * | * | * | ** | * | n/a | n/a | 7 | High |
| Strath et al. 2008 | * | * | * | * | ** | * | n/a | n/a | 7 | High |
| Swartz et al. 2003 | - | * | - | * | - | * | - | * | 4 | Low |
| Thompson et al. 2004 | - | * | - | * | * | * | n/a | n/a | 4 | High |
| Tolonen et al. 2018 | * | * | * | * | ** | * | n/a | n/a | 7 | High |
| Tucker et al. 2003 | - | * | * | * | - | * | n/a | n/a | 4 | Low |
| Tudor-Locke et al. 2009 | * | * | * | * | - | * | n/a | n/a | 5 | High |
| Vella et al. 2011 | * | * | * | * | - | * | n/a | n/a | 5 | High |
| Vella et al. 2009 | * | * | * | * | ** | * | n/a | n/a | 7 | High |
| Van Dyck et al. 2015 | * | * | * | * | ** | * | n/a | n/a | 7 | High |

Notes: Representativeness of the exposed cohort: One star = truly/somewhat representative of general female adults; Selection of the non-exposed cohort: One star = drawn from the same community as the exposed cohort; Comparability: One star = study controls for age, 2nd star = study controls for other confounders; Follow-up long enough for outcomes to occur: One star = ≥12 months; Adequacy of follow up: Complete follow up/all groups had similar loss to follow-up/less than 5% of total lost to follow-up

n/a: not applicable

**Supplementary Table B.2 Assessment of methodological quality of randomized studies (the Cochrane Collaboration’s tool).**

| Study | Random sequence generation | Allocation concealment | Blinding of participants and personnel | Blinding of outcome assessment | Incomplete outcome data | Selective reporting | Other sources of bias | Overall Quality |
| --- | --- | --- | --- | --- | --- | --- | --- | --- |
| Bailey et al. 2019 | L | L | L | L | L | L | L | High |
| Cayir et al. 2015 | L | U | L | L | L | L | L | U |
| Holliday et al. 2018 | L | L | L | L | L | L | L | High |
| Hornbuckle et al. 2012 | L | L | L | L | L | L | L | High |
| Moreau et al. 2001 | U | L | L | L | L | L | L | U |
| Pal et al. 2011 | U | L | L | L | L | L | L | U |
| Sugawara et al. 2006 | U | L | L | L | L | L | L | U |

Note: L, low risk; H, high risk, U, unclear.

**Supplementary Table B.3 GRADE assessment**

| **PA measures** | **No. of participants (#studies)** | **Study design** | **Quality Assessment** | | | | | **Absolute effect** | **Quality** |
| --- | --- | --- | --- | --- | --- | --- | --- | --- | --- |
|  |  |  | **Risk of bias^1^** | **Inconsistency^2^** | **Indirectness^3^** | **Inprecision^4^** | **Publication bias^5^** |  |  |
| **Steps** | 1970(11) | cross-sectional | no serious | no serious | no serious | no serious | no serious | BMI: favorable (9/10); null (1/10); %BF: favorable (5/5); WC: favorable (4/7); null (3/7); FM: favorable (2/2); VAT: favorable (1/1). | Low |
|  | 272(5) | RTs | no serious | serious | no serious | serious | not applicable | BMI: favorable (1/4); null (3/4); %BF: favorable (1/4); null (3/4); WC: favorable (1/3); null (2/3); FM: null (2/2); VAT: null (1/1). | Low |
|  | 147(3) | NRTs | no serious | serious | no serious | serious | not applicable | BMI: favorable (2/3); null (1/3); %BF: favorable (1/2); null (1/2); WC: favorable (2/3); null (1/3); FM: favorable (1/1); VAT: favorable (1/1). | Very low |
| **TPA** | 4158(7) | cross-sectional | no serious | no serious | no serious | no serious | not applicable | BMI: favorable (1/2); null (1/2); %BF: favorable (3/4); null (1/4); WC: favorable (2/2); VAT: favorable (2/1). | Low |
| **LPA** | 931(5) | cross-sectional | no serious | no serious | no serious | no serious | not applicable | BMI: null (2/2); %BF: null (2/2); WC: null (1/1); FM: null (1/1); VAT: null (1/1). | Low |
|  | 228(1) | Longitudinal | no serious | no serious | no serious | serious | not applicable | %BF:null (1/1). | Very low |
| **MPA** | 1095(5) | cross-sectional | serious | serious | no serious | no serious | not applicable | BMI: favorable (2/3); null (1/3); %BF: favorable (1/2); null (1/2); WC: favorable (2/3); null (1/3); FM: favorable (1/1); VAT: favorable (1/1). | Very low |
|  | 228(1) | Longitudinal | no serious | no serious | no serious | serious | not applicable | %BF: null (1/1). |  |
|  | 75(2) | RTs | no serious | no serious | no serious | serious | not applicable | BMI: null (1/1); WC: null (1/1); FM: null (1/1); VAT: null (1/1). | Moderate |
| **MVPA** | 5745(10) | cross-sectional | no serious | serious | no serious | serious | no serious | BMI: favorable (2/5); null (3/5); %BF: favorable (2/3); null (1/3); WC: favorable (2/4); null (2/4); FM: null (1/1); VAT: favorable (1/3); null (2/3). | Very low |
| **VPA** | 970(5) | cross-sectional | serious | no serious | no serious | no serious | not applicable | BMI: favorable (2/2); %BF: favorable (3/3); WC: favorable (1/1); FM: favorable (1/1); VAT: favorable (1/1). | Very low |
|  | 228(1) | Longitudinal | no serious | no serious | no serious | serious | not applicable | %BF: favorable (1/1). | Low |
|  | 17(1) | RTs | no serious | no serious | no serious | serious | not applicable | BMI: favorable effect (1/1). | Moderate |
| **10min PA bout** | 1854(3) | cross-sectional | no serious | serious | no serious | no serious | not applicable | BMI: favorable (1/2); null (1/2); WC: favorable (1/3); null (2/3); | Very low |

**Note:**

1. We downgraded one level if 50% to 75% of studies were at low quality and two levels if more than 75% of studies low quality.
2. We downgraded one level if findings were highly inconsistent.
3. We downgraded one level for indirectness if studies provided indirect evidence or made indirect comparisons.
4. We downgraded one level for imprecision if the number of participants was less than 400.
5. Publication bias test was not applicable when n<10.

BMI, body mass index; FM, fat mass; LPA, low intensity physical activity; MPA, moderate intensity physical activity; MVPA, moderate to vigorous intensity physical activity; PA, physical activity; RTs, random trials; TPA, total physical activity; VAT, visceral adipose tissue; VPA, vigorous physical activity; WC, waist circumference; %BF, percentage body fat.

**Supplementary Table C:** Characteristics of participants

| **Reference** | **Country** | **Race** | **Sample size** | **Age** | **BMI** | **Menstrual status** | **Diet** | **Education** | **Lifestyle** | **Socio-economic level** | **Tobacco** |
| --- | --- | --- | --- | --- | --- | --- | --- | --- | --- | --- | --- |
| **Ayabe et al. 2013** | Japan | Asian | 42 | 50±6 | 22.3±3.2 | n/r | n/r | n/r | physically inactive | part-time | n/r |
| **Bailey et al. 2007** | USA | Caucasian (96%) | 228 | 40.0±3.0 | 23.8±3.4 | premenopausal | n/r | college or more (57%) | n/r | n/r | non-smoker |
| **Bailey et al. 2014** | USA | Caucasian (90%) | 186 | 23.0±1.7 | 22.5±3.0 | n/r | no affected medications | n/r | n/r | collage student | n/r |
| **Bailey et al. 2015** | USA | Caucasian (88%) | 343 | 20.2±1.6 | 22.5±2.9 | n/r | no affected medications | n/r | n/r | n/r | non-smoker |
| **Bailey et al. 2019** | USA | n/r | 92 | 18.1±0.3 | 23.1±2.5 | n/r | no affected medications | college | physically inactive | collage student | n/r |
| **Cayir et al. 2015** Pedometer | Turkey | n/r | 45 | 41.1±9.3 | 35.9±4.5 | n/r | n/r | n/r | n/r | employed (43%) | n/r |
| **Cayir et al. 2015** Control | Turkey | n/r | 39 | 38.8±11.2 | 34.3±2.8 | n/r | n/r | n/r | n/r | employed (33%) | n/r |
| **de Hoed et al. 2008** | Netherlands | n/r | 80 | 21.0±2.0 | 21.8±2.5 | n/r | no affected medications | n/r | n/r | collage student | n/r |
| **Diniz et al. 2015** Active | Brazil | n/r | 25 | 55.8±7.2 | 26.9±5.1 | postmenopausal | no affected medications | n/r | physically active | n/r | n/r |
| **Diniz et al. 2015** Sedentary | Brazil | n/r | 24 | 61.6±6.2 | 29.1±9.0 | postmenopausal | no affected medications | n/r | physically inactive | n/r | n/r |
| **Graff et al. 2012** | Brazil | Caucasian (73%) | 68 | 28.0±6.0 | 28.0±6.0 | premenopausal | no affected medications | n/r | n/r | n/r | n/r |
| **Green et al. 2014** | USA | Caucasian (92%) | 50 | 24.0±4.8 | 27.0±4.8 | premenopausal | no affected medications | collage student (84%) | n/r | collage student (84%) | no smoke for 6 months |
| **Hasan et al. 2018** | UAE | n/r | 52 | 21.43±4.8 | 27.5±5.6 | n/r | no affected medications | college | n/r | college student | n/r |
| **Holliday et al. 2018** | UK | n/r | 58 | 41.0±2.0 | 29.2±3.4 | n/r | no affected medications | n/r | physically inactive | n/r | non-smoker |
| **Hornbuckle et al. 2005** | USA | African American | 69 | 51.4±5.4 | 30.9±6.8 | n/r | n/r | n/r | n/r | n/r | non-smoker |
| **Hornbuckle et al. 2012** | USA | African American | 44 | 49.0±5.5 | 34.7±6.4 | n/r | n/r | n/r | physically inactive | n/r | no smoke for 6 months |
| **Koniak-Griffin et al. 2014** | USA | Latina | 210 | 44.6±7.9 | 32.6±5.7 | n/r | n/r | n/r | n/r | low income | n/r |
| **Moreau et al. 2001** | USA | n/r | 24 | 54.0±1.0 | n/r | postmenopausal | n/r | n/r | physically inactive | n/r | non-smoker |
| **Musto et al. 2010** Active | USA | n/r | 43 | 46.3±10.4 | 30.4±5.5 | n/r | n/r | n/r | physically inactive | n/r | n/r |
| **Musto et al. 2010** Control | USA | n/r | 34 | 45.7±9.5 | 29.5±5.0 | n/r | n/r | n/r | physically inactive | n/r | n/r |
| **Pal et al. 2011** 10000 steps | Australia | n/r | 13 | 41.4±2.7 | 28.9±1.2 | n/r | no affected medications | n/r | physically inactive | n/r | non-smoker |
| **Pal et al. 2011** 30 minutes | Australia | n/r | 15 | 45.3±2.2 | 29.7±1.1 | n/r | no affected medications | n/r | physically inactive | n/r | non-smoker |
| **Panton et al. 2007** | USA | African American | 35 | 48±8 | 42.3±9.8 | n/r | no affected medications | n/r | n/r | low income | non-smoker (83%) |
| **Park et al. 2011** | Japan | Asian | 100 | 51.8±11.2 | 23.5±4.4 | n/r | n/r | n/r | n/r | n/r | n/r |
| **Pelclová et al. 2012** Czech Republic | Czech Republic | n/r | 45 | 64.2±3.8 | 26.1±3.6 | n/r | n/r | n/r | n/r | the Third Age University student | n/r |
| **Pelclová et al. 2012** Slovakia | Slovakia | n/r | 51 | 61.8±5.0 | 27.5±4.2 | n/r | n/r | n/r | n/r | the Third Age University student | n/r |
| **Pelclová et al. 2012** Poland | Poland | n/r | 71 | 62.5±5.2 | 27.9±4.5 | n/r | n/r | n/r | n/r | the Third Age University student | n/r |
| **Phelan et al. 2007** weight-loss maintainers | USA | Caucasian (93%) | 135 | 49.1±11.3 | 22.0±1.6 | n/r | n/r | college or more (76%) | n/r | n/r | n/r |
| **Phelan et al. 2007** always normal weight | USA | Caucasian (95%) | 102 | 48.6±11.2 | 21.1±1.3 | n/r | n/r | college or more (80%) | n/r | n/r | n/r |
| **Slater et al. 2021** Pacific normal weight | New Zealand | Pacific | 61 | 25.0±7.0 | 25.9±3.9 | premenopausal | n/r | n/r | n/r | low income | n/r |
| **Slater et al. 2021** Pacific obesity | New Zealand | Pacific | 55 | 26.0±.0 | 35.6±6.1 | premenopausal | n/r | n/r | n/r | more deprived | n/r |
| **Slater et al. 2021** European normal weight | New Zealand | European | 85 | 22.5±2.1 | 22.5±2.1 | premenopausal | n/r | n/r | n/r | less deprived | n/r |
| **Slater et al. 2021** European obesity | New Zealand | European | 74 | 33.7±3.8 | 33.7±3.8 | premenopausal | n/r | n/r | n/r | less deprived | n/r |
| **Smith et al. 2013** | USA | Caucasian (81%) | 183 | 38.2±5.8 | 32.6±4.0 | n/r | n/r | college or more (71%) | physically inactive | n/r | n/r |
| **Sternfeld et al. 2005** Chinese premenopausal | USA | Asian | 78 | 49.7±2.1 | n/r | premenopausal | n/r | n/r | n/r | n/r | n/r |
| **Sternfeld et al. 2005** Chinese postmenopausal | USA | Asian | 79 | 52.3±2.4 | n/r | postmenopausal | n/r | n/r | n/r | n/r | n/r |
| **Sternfeld et al. 2005** Caucasian premenopausal | USA | Caucasian | 45 | 49.4±1.9 | n/r | premenopausal | n/r | n/r | n/r | n/r | n/r |
| **Sternfeld et al. 2005** Caucasian postmenopausal | USA | Caucasian | 46 | 52.7±2.8 | n/r | postmenopausal | n/r | n/r | n/r | n/r | n/r |
| **Strath et al. 2008** | USA | Caucasian (75%) | 1594 | 48.1±17.1 | 28.4±6.8 | n/r | n/r | n/r | n/r | n/r | non-smoker(80%) |
| **Sugiura et al. 2002** intervention | Japan | Asian | 14 | 48.6±4.2 | 22.3±1.6 | n/r | no affected medications | n/r | physically inactive | n/r | n/r |
| **Sugiura et al. 2002** control | Japan | Asian | 13 | 48.0±3.6 | 22.6±1.9 | n/r | no affected medications | n/r | physically inactive | n/r | n/r |
| **Swartz et al. 2003** | USA | n/r | 18 | 53.3±7.0 | 35.0±5.1 | n/r | n/r | n/r | physically inactive | n/r | non-smoker |
| **Thompson et al. 2004** | USA | n/r | 80 | 50.3±6.8 | 26.0±5.1 | n/r | n/r | n/r | n/r | n/r | n/r |
| **Tolonen et al. 2018** | Finland | n/r | 837 | 31-46 | 24.7-26.1 | n/r | n/r | n/r | n/r | n/r | n/r |
| **Tucker et al. 2003** | USA | Caucasian (90%) | 278 | 40.1±3.0 | 23.9±3.3 | premenopausal | n/r | college or more (37%) | n/r | n/r | non-smoker |
| **Tudor-Locke et al. 2009** | Australia | n/r | 158 | 56.4±1.4 | 26.9±5.4 | n/r | n/r | college or more (46%) | n/r | employed (63%) | n/r |
| **Van Dyck et al. 2015** | multi-country | n/r | 3027 | 18-65 | 22.6-28.0 | n/r | n/r | n/r | n/r | n/r | n/r |
| **Vella et al. 2009** | USA | Hispanic | 60 | 24.9±0.7 | 23.6±0.5 | n/r | no affected medications | n/r | n/r | n/r | no smoke for 6 months |
| **Vella et al. 2011** no meet PA Guideline | USA | Hispanic | 42 | 25.2±5.6 | 23.8±4.0 | n/r | no affected medications | n/r | n/r | n/r | no smoke for 6 months |
| **Vella et al. 2011** meet PA Guideline | USA | Hispanic | 18 | 24.4±4.9 | 23.0±4.6 | n/r | no affected medications | n/r | n/r | n/r | no smoke for 6 months |

Note: n/r, not report; PA, physical activity

**Supplementary Table D:** Ascertainment and measurement characteristics of objectively measured PA

| **Reference** | **Divice name** | **Wearing position** | **Frequency/Epoch** | **Required time** | **Valid time** | **Reported measure, Cut-off ponits/definitions** |
| --- | --- | --- | --- | --- | --- | --- |
| **Ayabe et al. 2013** | A: Lifecorder-Ex,uniaxial | left waist | 32Hz/4s | 10d/wake | 10h/7d | MVPA min/d, 4-9METs;  VPA min/d, 7-9METs;  TPA min/d, 1-9METs |
| **Bailey et al. 2007** | A: ActiGraphs, uniaxial | left hip | 10 min | 7d/day exp. w | / | LPA group, less than 30,000 counts per epoch;  MPA, 30000-50000 counts per epoch;  VPA, ≥ 50000 counts per epoch |
| **Bailey et al. 2014** | P: Omeron HJ-720-ITC | right-waist/blinded | / | 7d/day exp. w | 80% time(7a.m.-11p.m.)/7d | Aerobic steps n/d, 60 steps per minute for a minimum of 10 minutes |
| **Bailey et al. 2015** | A: ActiGraph GT3x, triaxial | right hip | 60s | 7d/day exp. w | 75% (7a.m.-11p.m.) | LPA min/d, 250-2019 cpm;  MPA min/d, 2020-5999 cpm;  VPA min/d, ≥ 6000 cpm;  MVPA min/d, ≥ 2020 cpm |
| **Bailey et al. 2019** | A: ActiGraph GT3x, triaxial;  P: Omeron HJ-720-IT | hip | 60s | 4d (2weekday, 2weekend)/day exp. W; 24w/day exp. W | 75% (7a.m.-11p.m.) | LPA min/d, 100-2019 cpm;  MVPA min/d, ≥ 2020 cpm |
| **Cayir et al. 2015** | P: Voit 3d | / | / | / | / | / |
| **de Hoed et al. 2008** | A: Tracmor IV, triaxial | / | / | 14d/wake exp. w | / | TPA, megacounts/day |
| **Diniz et al. 2015** | A: ActiGraph GT3x, triaxial | waist | 60s | 7d/wake exp. w | 10h/5d | LPA, <1952 cpm;  MPA, 1952-5724 cpm;  VPA, 5725-9498 cpm;  VVPA, >9499 cpm |
| **Graff et al. 2012** | P: BP 148 | / | / | 6d/day exp. w | / | inactive, <6000 step/d;  active, ≥ 6000 step/d |
| **Green et al. 2014** | A: ActiGraph GT3X+, triaxial | right hip | 60s | 7d/day exp. w | 10h/4d(1 weekend) | LPA, 150-2689 cpm;  MVPA, ≥2690 cpm |
| **Hasan et al. 2018** | P: KenzLifeCoder e-step | waist | / | 9w/wake exp. w | / | sedentary, <5000 steps/d;  low active, 5000-7499 steps/d;  somewhat active, 7500-9999 steps/d;  active, 10000-12499 steps/d;  highly active, ≥12500 steps/d |
| **Holliday et al. 2018** | A: ActiGraph GT3X+, triaxial | right hip | 15s | 3d/wake | 10h/3d | MPA min/d, 2020-5999 cpm;  VPA min/d, ≥ 6000 cpm;  MVPA min/d, ≥ 2020 cpm |
| **Hornbuckle et al. 2005** | P: New Lifestyles Digi-Walker SW-200 | hip | / | 7d/wake | 7d | sedentary, <5000 steps/d;  low active, 5000-7499 steps/d;  somewhat active, 7500-9999 steps/d;  active, ≥10000 steps/d |
| **Hornbuckle et al. 2012** | P: New Lifestyles Digi-Walker SW-200 | hip | / | / | / | / |
| **Koniak-Griffin et al. 2014** | A: Kenz Lifecorder Plus, uniaxial |  | 4s | 7d/wake exp. w | 8h/4d | MVPA, ≥3METS |
| **Moreau et al. 2001** | P: Yamax SW200 pedometer | waist | / | 1-2w/wake | / | / |
| **Musto et al. 2010** | P: Sportline 330 | / | / | 7d/wake | / | / |
| **Pal et al. 2011** | P: Yamax Digi-Walker SW-200 | waist | / | / | / | / |
| **Panton et al. 2007** | P: Yamax Digi-Walker SW-200, sealed | waist | / | 2w/wake exp. w | / | Sedentary, <5000 steps/d;  Active, ≥5000 steps/d |
| **Park et al. 2011** | A: Lifecorder EX, uniaxial | left waist | / | / | / | LPA, <3 METs;  MPA, 3-6 METs;  VPA, ≥6 METs |
| **Pelclová et al. 2012** | A: ActiGraph GT1M, uniaxial | right hip | 60s | 8d/wake exp. w | 10h/7d | LPA, <1952 cpm;  MPA, 1952-5724 cpm;  VPA, >5724 cpm |
| **Phelan et al. 2007** | A: RT3, triaxial | waist | 60s | wake | 2h/4d | LPA, ≥2METs;  MPA, ≥3METs;  VPA, ≥5METs |
| **Slater et al. 2021** | A: Actigraph w-GT3X, triaxial; A: Acti-Watch | non-dominant hip; non-dominant wrist | 60s | 8d/day exp. w | 12h/4d | sedentary, 0-99 cpm;  LPA, 100-2019 cpm;  MPA, 2020-5998 cpm;  VPA, ≥5999 cpm;  MVPA, ≥2020 cpm |
| **Smith et al. 2013** | A: Actigraph AM7164, uniaxial | dominant hip | 60s | 7d/wake | 10h/4d | LPA, 100-1951 cpm;  MPA, 1952-5724 cpm;  VPA, ≥5725 cpm;  TPA, ≥100 cpm;  sedentary, 0-99 cpm |
| **Sternfeld et al. 2005** | A: CSA; uniaxial | waist | / | 7d/wake exp. w | / | TPA, mean cpm MPA, 1000-4999 cpm VPA, ≥5000 cpm |
| **Strath et al. 2008** | A: AM-7164; uniaxial | right hip | 60s | 7d | 10h/4d | MVPA, ≥760 cpm |
| **Sugawara et al. 2006** | A: Lifecorder, uniaxial | hip | 32Hz/4s | 14d | 7d | LPA, <4METs;  MPA, 4-6 METs;  VPA, >6 METs |
| **Swartz et al. 2003** | P: Yamax Digi-Walker SW-200, | / | / | 12w | / | / |
| **Thompson et al. 2004** | P: Yamax Digi-Walker SW-200 | right waist | / | 7d/wake exp. w | 7d | inactive, < 6000 steps;  somewhat active, 6000-9999 steps; regularly active, ≥10000 steps |
| **Tolonen et al. 2018** | P: Walking style One, HJ-152R-E | waist | / | 7d/day exp. w | 8h/4d | / |
| **Tucker et al. 2003** | A: CSA; uniaxial TPA (counts) (min/w) Intensity of PA (counts/10min) | left hip | 10 min | 7d/day exp. w | / | TPA, counts 10000-19999 counts, -2.7 METs 20000 to 49999 counts, -4.2 METs 50000 and more, -8.5+ METs |
| **Tudor-Locke et al. 2009** | P: Yamax Digiwalker SW700 | waist | / | 7d/day exp. w | / | / |
| **Van Dyck et al. 2015** | A: ActiGraph 7164/71256, GT1M, ActiTrainer, GT3X; uniaxial&triaxial | right hip | 60s | 7d/wake exp. w | / | LPA, 100-1952 cpm;  MPA, 1952-5724 cpm;  VPA, >5725 |
| **Vella et al. 2009** | A: Actigraph GT1M, uniaxial | right hip | 60s | 4d(3weekday, 1weekend)/wake exp. w | 12h/4d | / |
| **Vella et al. 2011** | A: Actigraph GT1M, uniaxial | right hip | 60s | 4d(3weekday, 1weekend)/wake exp. w | 12h/4d | LPA, 100-1951 cpm;  MPA, 1952-5724 cpm;  VPA, ≥5725 cpm |

Note: A, accelerometer; cpm, counts per minute; d, day; exp, expect; h, hour; LPA, light intensity physical activity; METs, metabolic equivalents; MPA, moderate intensity physical activity; MVPA, moderate to vigorous intensity physical activity; P, pedometer; VPA, vigorous intensity physical activity; W, water activities
